# Genome-wide characterization of *SPL* family in *Medicago truncatula* reveals the novel roles of *miR156*/*SPL* module in spiky pod development

**DOI:** 10.1186/s12864-019-5937-1

**Published:** 2019-07-05

**Authors:** Hongfeng Wang, Zhichao Lu, Yiteng Xu, Lingcui Kong, Jianjun Shi, Yafei Liu, Chunxiang Fu, Xiaoshan Wang, Zeng-Yu Wang, Chuanen Zhou, Lu Han

**Affiliations:** 1 The Key Laboratory of Plant Development and Environmental Adaptation Biology, Ministry of Education, School of Life Science, Shandong University, Qingdao, 266101 China; 2 School of Life Science, Guangzhou University, Guangzhou, 510006 China; 3 Key Laboratory of Biofuels, Shandong Provincial Key Laboratory of Energy Genetics, Qingdao Institute of Bioenergy and Bioprocess Technology, Chinese Academy of Sciences, Qingdao, 266101 China; 4 Department of Grassland Science, College of Animal Science and Technology, Yangzhou University, Yangzhou, 225009 China; 5 Noble Research Institute, LLC, Ardmore, OK USA; 6 Present Address: Grassland Agri-Husbandry Research Center, Qingdao Agricultural University, Qingdao, People’s Republic of China

**Keywords:** *SPL* genes, *miR156*, Seed development, Spiky pod formation, *Medicago truncatula*, Legume

## Abstract

**Background:**

SQUAMOSA Promoter Binding Protein-Likes (SPLs) proteins are plant-specific transcription factors that play many crucial roles in plant growth and development. However, there is little information about *SPL* family in the model legume *Medicago truncatula*.

**Results:**

In this study, a total of 23 *MtSPL* genes were identified in *M. truncatula* genome, in which 17 of the *MtSPLs* contained the putative *MtmiR156* binding site at the coding or 3′ UTR regions. Tissue-specific expression pattern analysis showed that most *MtmiR156*-targeted *MtSPLs* were highly expressed in seed and pod. The observation of *MtmiR156B*-overexpressing plants reveals that *MtmiR156/MtSPL* modules are not only involved in the development of leaves and branches, but also in the seed pod development, especially the formation of spine on pod.

**Conclusion:**

The spines on pods are developed in many plant species, which allow pods to adhere to the animals, and then be transported on the outside. This study sheds light on the new function of *SPL* family in seed dispersal by controlling the formation of spiky pod, and provides insights on understanding evolutionary divergence of the members of *SPL* gene family among plant species.

**Electronic supplementary material:**

The online version of this article (10.1186/s12864-019-5937-1) contains supplementary material, which is available to authorized users.

## Background

Transcription factors (TFs) play an essential role in regulatory networks of many important developmental processes by activating or repressing the transcription of downstream target genes [1]. The SQUAMOSA Promoter-Binding Protein-Likes (SPLs) proteins are plant-specific TFs and all of them have a highly conserved SBP domain with proximately 78 amino acids in length [2–4]. The SBP domain contains two zinc-finger like structure (Cys-Cys-Cys-His and Cys-Cys-His-Cys) and one nuclear localization signal (NLS) motif [5, 6]. SPL proteins specifically recognize and bind to the cis-element TNCGTACAA, which is located at the promoters of target genes with GTAC as its core sequence [5, 7]. The initial two *SPL* genes, *AmSBP1* and *AmSBP2*, are firstly identified from the *Antirrhinum majus*, and they act as transcriptional activators and regulate the expression of floral meristem identity gene *SQUAMOSA* [3]. The *SPL* genes have been reported to play the important role in regulation of multiple aspects of plant growth and development, including leaf and shoot development [8, 9], vegetative phase change [10, 11], flowering [12], sporogenesis [13], male fertility [14], plant hormone signal transduction [15], and copper homeostasis [16]. So far, the *SPL* families have been identified and characterized in several plant species, such as *Arabidopsis thaliana* [2], *Oryza sativa* [17], *Solanum lycopersicum* [18], *Gossypium raimondii* [19], *Vitis vinifera* [20], *Brassica napus* [21], *Glycine max* [22], *Prunus mume* [23], *Arachis hypogaea* L. [24], *Phyllostachys edulis* [25] and *Capsicum annuum* L. [26].

Many microRNAs have been identified, which regulate gene expression by binding and cleaving their target mRNAs [27, 28]. *miRNA156* is one of the highly conserved miRNA families in plants [29]. In *Arabidopsis*, in total 16 *SPLs* are identified and termed as *AtSPL1* to *AtSPL16*, respectively [5]. Among them, 10 are targets of *AtmiR156*, which are *AtSPL2*-*AtSPL6*, *AtSPL9*-*AtSPL11*, *AtSPL13*, and *AtSPL15* [9, 12, 30–32]. In rice, 19 *OsSPL* genes have been identified, and 11 of them are targeted by *OsmiR156* [17]. Most *Arabidopsis miR156* binding sites of the targeted-*SPL* genes are located in the downstream of the SBP domain at the coding sequences, while in *AtSPL3*, *AtSPL4*, and *AtSPL5*, they are located in the 3′ UTR of the mRNAs [12].

These *miR156*-targeted *SPL* genes play redundant roles in plant morphology and development among different species. Several studies show that *SPL* genes are involved in the regulation of flower and inflorescence development. In *Arabidopsis*, *AtSPL3*, *AtSPL4* and *AtSPL5* have similar functions and play vital roles in vegetative phase change and floral transition [7, 9, 12]. Furthermore, *AtSPL3* can bind directly on the promoter regions of *AP1*, *LFY* and *FUL* to activate their expression in controlling the timing of flower formation [33]. In addition, *SOC1* and *FT* regulate the expression of *AtSPL3*, *AtSPL4* and *AtSPL5* in response to photoperiod and gibberellin (GA) signals to promote flowering [34]. In wheat, two paralogous *SPL* genes, *TaSPL20* and *TaSPL21*, are highly expressed in the lemma and palea, and ectopic expression of them in rice can promote panicle branching [35]. *SPL* genes also play important roles in regulating lateral organ and shoot development. In *Arabidopsis*, *AtSPL2*, *AtSPL10* and *AtSPL11* redundantly regulate proper development of lateral organs in association with shoot maturation in the reproductive phase [36]. Besides, mutant phenotype analysis shows that *AtSPL9* and *AtSPL15* act redundantly in regulation of the juvenile-to-adult phase transition [11]. In maize, *SPL* transcription factor *TASSELSHEATH4* plays an essential role in bract development and the establishment of meristem boundaries [37]. Two duplicated loci, *UNBRANCHED2* and *UNBRANCHED3*, affect crop productivity traits by regulating the rate of lateral primordia initiation [38]. In addition, a series of studies show that *SPL* genes are involved in the regulation of seed and fruit development. In rice, the *OsmiR156*-targeted *SPL* gene, *OsSPL14*/*IDEAL PLANT ARCHITECTURE 1* (*IPA1*), regulates shoot branching in the vegetative stage and enhances rice grain yield at the productive stage [39, 40]. *OsSPL16*/*GRAIN WIDTH 8* (*GW8*) acts as a positive regulator of cell proliferation and is involved in control of grain size, shape and quality [41]. In tomato, the *SPL* gene *COLORLESS NON-RIPENING* (*CNR*) is critical for normal fruit ripening, and mutation in *CNR* results in colorless fruits with a substantial loss of cell-to-cell adhesion [42].

*Medicago truncatula* is a fast-emerging model legume for functional genomics study. However, the information and function of *SPL* gene family in *M. truncatula* are largely unknown. In this study, we reported the genome-wide identification and characterization of *SPL* genes in *M. truncatula*. Totally, 23 *MtSPL* genes were identified, and their phylogenetic relationship, protein motifs, gene structures, and chromosomal locations were analyzed. Furthermore, we found that most *MtmiR156*-targeted *MtSPL* genes were highly expressed in pod and seed. Overexpression of *MtmiR156B* transgenic plants displayed the small seeds and pods, especially the loss of pod spine. These findings demonstrate that *MtmiR156*-targeted *MtSPL* genes play the novel roles in pod and seed development in *M. truncatula*.

## Results

### Genome-wide identification of *MtSPL* genes

**Fig. 1:**
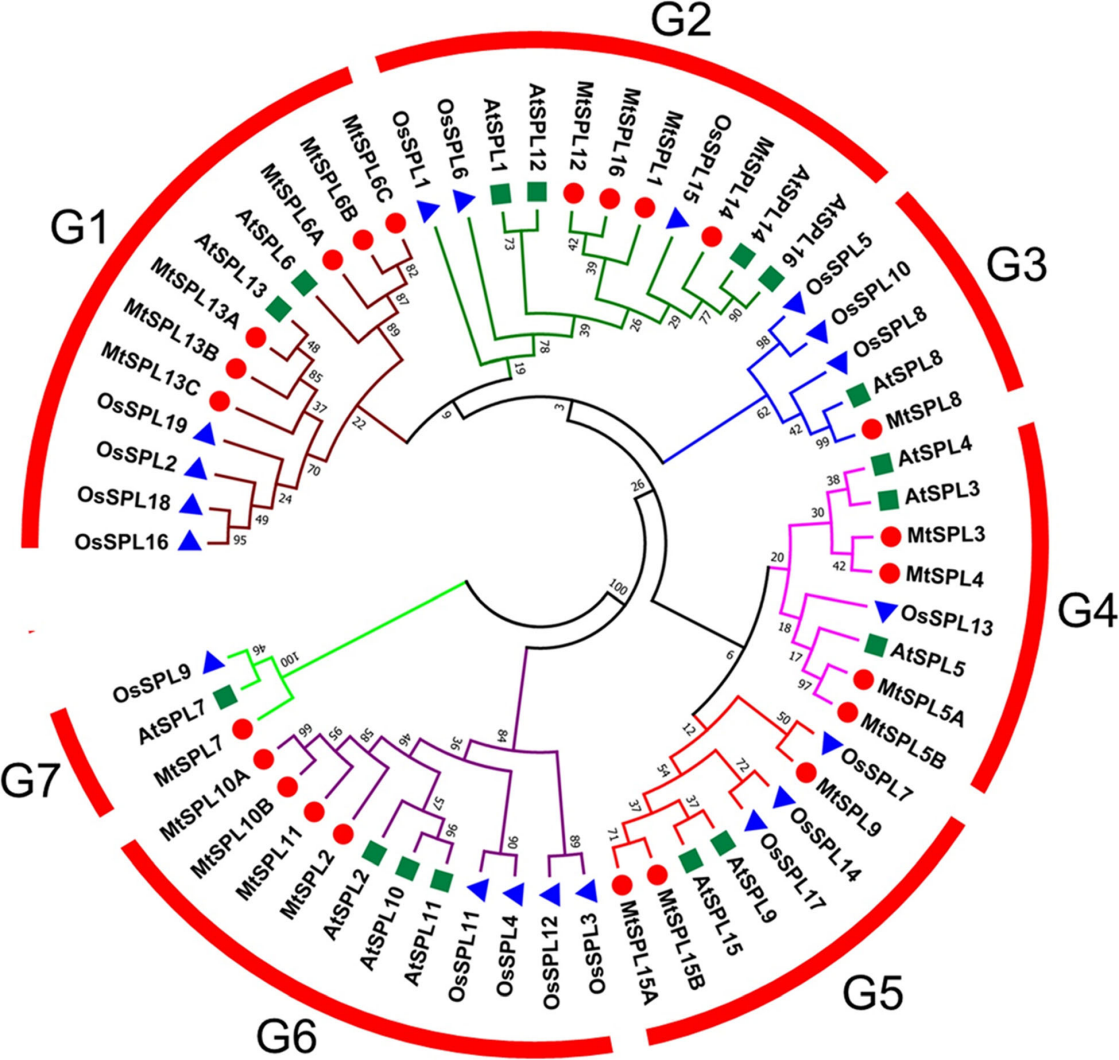
Phylogenetic analysis of SPL proteins from *M. truncatula*, *Arabidopsis* and rice. The Neighbor-Joining (NJ) phylogenetic tree was constructed using full length SPL protein sequences in MEGA 7.0 with 1000 replicates

In order to identify *SPL* genes in *M. truncatula* genome, we executed a genome-wide search of *MtSPLs* by protein BLAST using the 16 AtSPLs and 19 OsSPLs sequences against the *Medicago truncatula* Genome Database. Initially, a total of 24 putative *MtSPL* sequences were obtained. Medtr8g066240 was excluded from the *MtSPL* gene family due to the absence of a complete SBP domain. Therefore, totally 23 *MtSPLs* with the conserved SBP domain were characterized in genome of *M. truncatula* (Additional file 1). The *MtSPL* genes were named according to their closest *Arabidopsis* orthologs (Fig. 1). The protein lengths of *MtSPLs* ranged from 143 to 1025 amino acids, and the gene locus, isoelectric point, intron number, and chromosome location of the *MtSPL* genes were shown in Table 1.

**Table 1:** The *SPL* gene family in *M. truncatula*

Name	Gene ID	CDS (bp)	Introns	Length (aa)	pI	Location	miR156 target
*MtSPL1*	Medtr1g086250	3012	9	1003	5.99	chr1:38604282..38611936+	NO
*MtSPL2*	Medtr3g085180	1305	3	434	8.33	chr3:38492623..38497369+	YES
*MtSPL3*	Medtr4g088555	435	1	144	6.97	chr4:35174504..35179012-	YES
*MtSPL4*	Medtr2g014200	432	1	143	6.75	chr2:3964615..3967481+	YES
*MtSPL5A*	Medtr2g078770	516	1	171	8.31	chr2:32971840..32974296-	YES
*MtSPL5B*	Medtr8g463140	543	0	180	8.95	chr8:22196925..22198280-	YES
*MtSPL6A*	Medtr5g046670	1503	4	500	7.43	chr5:20459089..20465349-	YES
*MtSPL6B*	Medtr2g461920	1620	2	539	6.78	chr2:25606881..25611792+	YES
*MtSPL6C*	Medtr4g109770	1413	2	470	6.44	chr4:45646472..45650584-	YES
*MtSPL7*	Medtr2g020620	2238	10	745	6.63	chr2:6886415..6893572+	NO
*MtSPL8*	Medtr8g005960	984	0	327	8.48	chr8:419202..421415-	NO
*MtSPL9*	Medtr7g444860	945	0	314	8.58	chr7:15012335..15016587-	YES
*MtSPL10A*	Medtr8g080680	1197	0	398	7.55	chr8:34725302..34727462-	YES
*MtSPL10B*	Medtr8g080670	1239	0	412	7.93	chr8:34719933..34722421-	YES
*MtSPL11*	Medtr8g080690	1131	0	376	8.65	chr8:34729479..34731697-	YES
*MtSPL12*	Medtr7g110320	3006	0	1001	6.13	chr7:45210190..45220920-	NO
*MtSPL13A*	Medtr8g096780	1173	0	390	8.19	chr8:40622636..40626632+	YES
*MtSPL13B*	Medtr3g099080	1131	2	376	7.04	chr3:45410078..45413482+	YES
*MtSPL13C*	Medtr7g028740	1104	0	367	8.2	chr7:9871981..9875095+	YES
*MtSPL14*	Medtr1g035010	3012	8	1003	7.5	chr1:12692334..12698670+	NO
*MtSPL15A*	Medtr7g092930	1014	0	337	8.91	chr7:36893347..36897295-	YES
*MtSPL15B*	Medtr1g053715	1053	2	350	8.86	chr1:22678198..22682537-	YES
*MtSPL16*	Medtr2g046550	3078	9	1025	6.64	chr2:20453789..20462574+	NO

### Phylogenetic analysis and chromosomal localization of *MtSPL* genes

**Fig. 2:**
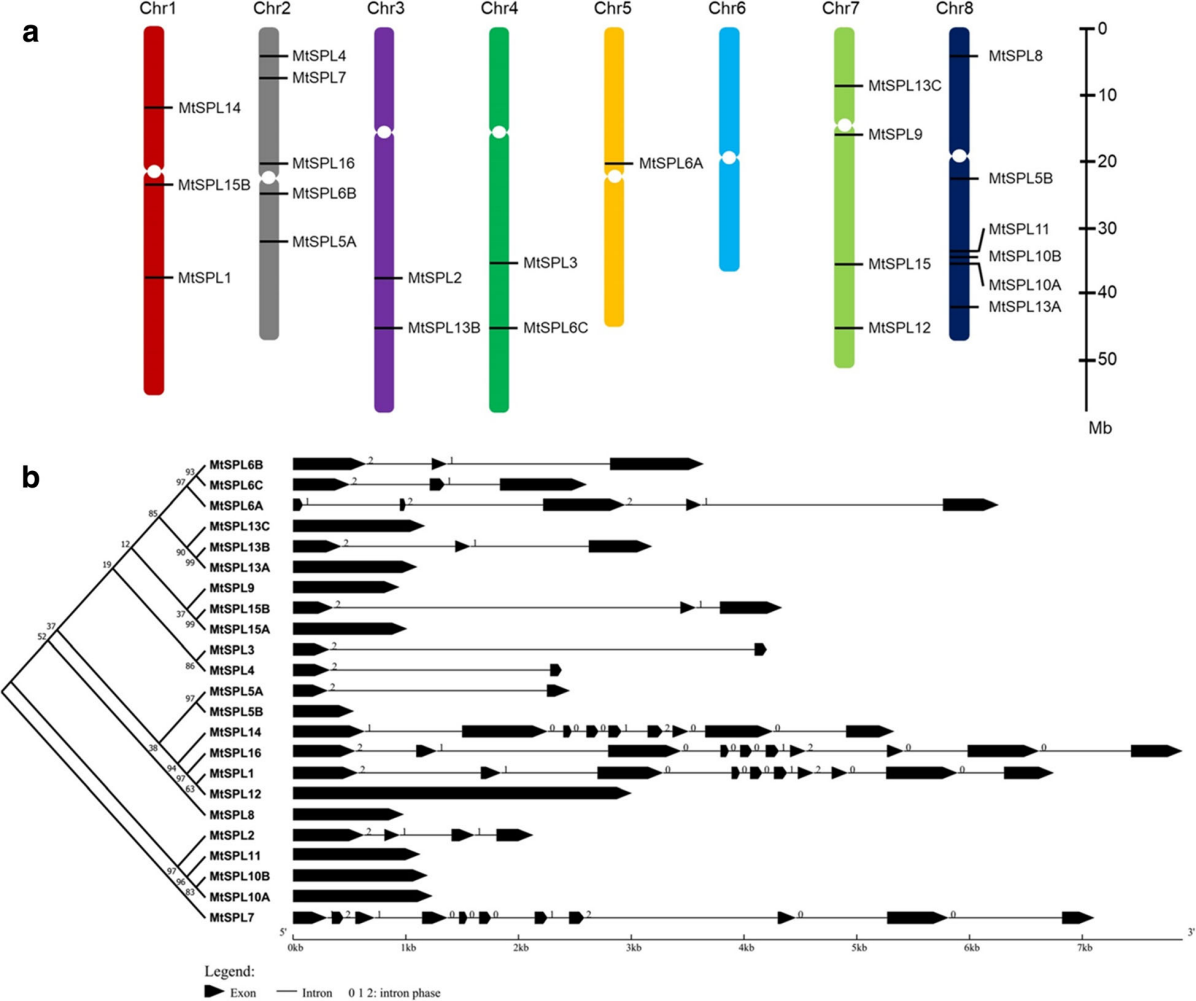
Chromosomal distribution, phylogenetic relationship and gene structure of *MtSPL* genes. **a** The distribution of *MtSPL* genes on *M. truncatula* chromosomes based on the genome annotation. The chromosomes are indicated with different colors. The scale (Mb) represents the lengths of the chromosomes. **b** Phylogenetic analysis and exon/intron structures of *MtSPL* genes. Exons and introns are indicated by black wedges and black lines. The scale (Kb) represents the lengths of the *MtSPL* genes

To further achieve the evolutionary relationship between *MtSPL* genes and other *SPLs*, a phylogenetic tree containing 16 AtSPLs, 19 OsSPLs, and 23 MtSPLs was constructed using MEGA7.0 with Neighbor-Joining method (Fig. 1). According to the phylogenetic analyses, the 58 SPL proteins were clustered into seven distinct groups (named G1-G7), and each group contained at least one SPL from *A. thaliana*, rice, and *M. truncatula* (Fig. 1). To determine the chromosome distribution of *MtSPL* genes in *M. truncatula*, the 23 *MtSPL* genes were located on each chromosome based on the *M. truncatula* genome data. These *MtSPL* genes showed uneven distribution on the *M. truncatula* chromosomes (Fig. 2a). Chromosome 8, 2, 7, and 1 contained 6, 5, 4, and 3 *MtSPL* genes, respectively. Both chromosome 3 and 4 contained 2 *MtSPL* genes, chromosome 5 contained only one *MtSPL* gene, and no *MtSPL* gene located on chromosome 6.

### Conserved motifs and gene structure analysis of *MtSPL* genes

**Fig. 3:**
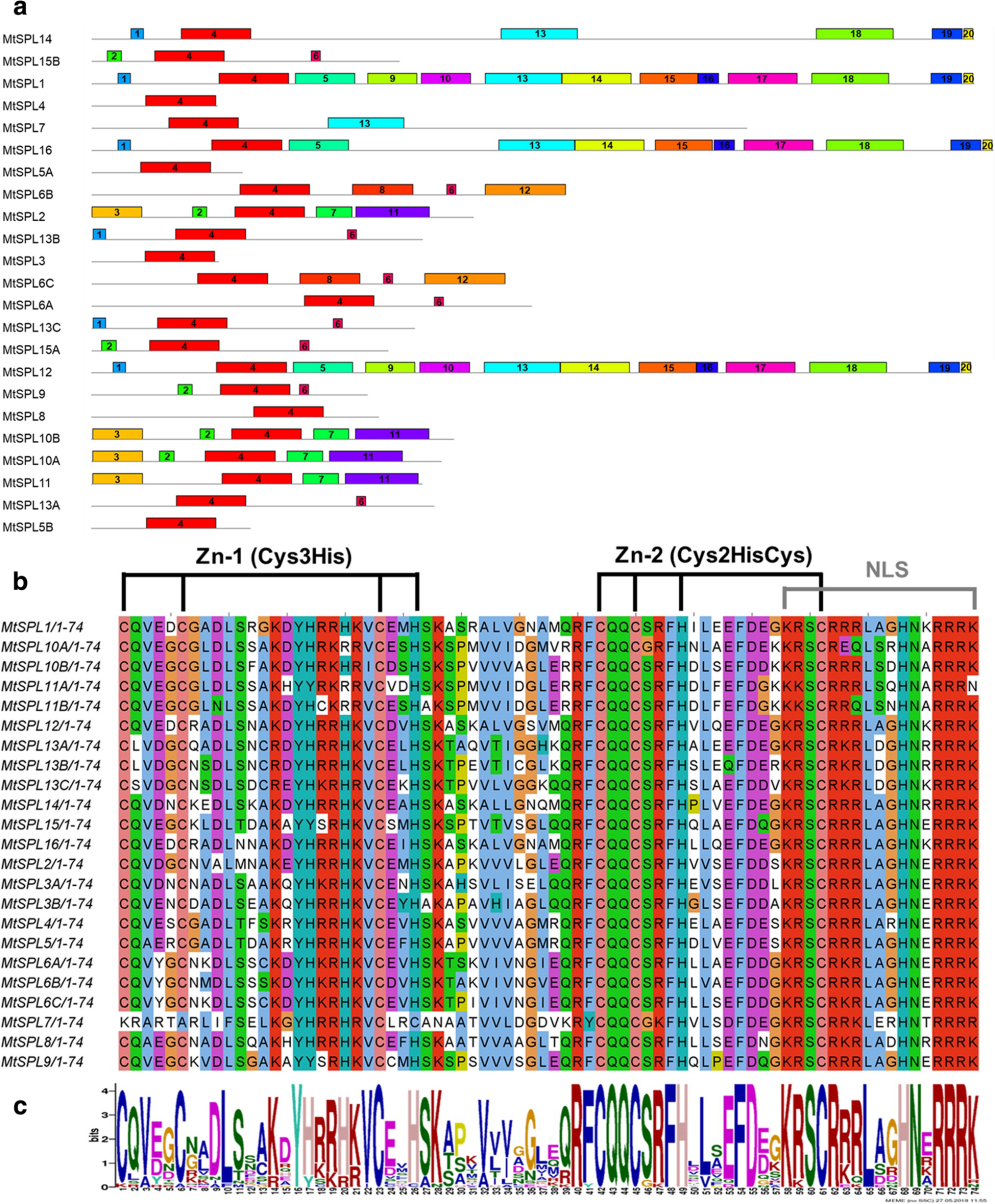
Conserved domains and motifs in MtSPL proteins*.* **a** The full length MtSPL protein sequences were used to execute the motif search on MEME tool. Domains and motifs were represented by the boxes with different numbers and colors. **b** Alignment of the conserved SBP domain in MtSPL proteins. Multiple SBP domain sequences alignment was performed using Jalview software. Two Zn-finger structures (Zn-1, Cys3His and Zn-2, Cys2HisCys) and one NLS are indicated. **c** Sequence logo of the conserved SBP domain of MtSPLs. Sequence logo was obtained from Weblogo online software. The overall height of each stack indicates the sequence conservation at each position, while the height of the letters within each stack indicates the relative frequency of the corresponding amino acid

To further understand the structural diversity of the *MtSPL* genes, gene exon/intron structure analysis was carried out using online Gene Structure Display Server tool. The exon/intron structures of the 23 *MtSPL* genes were generated by alignment of *MtSPL* gene coding sequences with their corresponding genomic sequences. Gene structure illustrations displayed the high variation in the number of introns, from 0 to 10 (Fig. 2b). To gain a better understanding of the protein sequence characteristics of the MtSPLs, online MEME search was performed to analyze the conserved domains and motifs. Besides the conserved SBP domain (motif 4), in total 20 conserved motifs were identified in MtSPLs (Fig. 3a, Additional file 2). The conserved SBP domain consisted of three motifs: zinc finger 1 (Zn-1, C3H), zinc finger 2 (Zn-2, C2HC), and a nuclear localization signal (Fig. 3b, c).

### Analysis of *MtmiR156* and its target sequences in *MtSPLs*

**Fig. 4:**
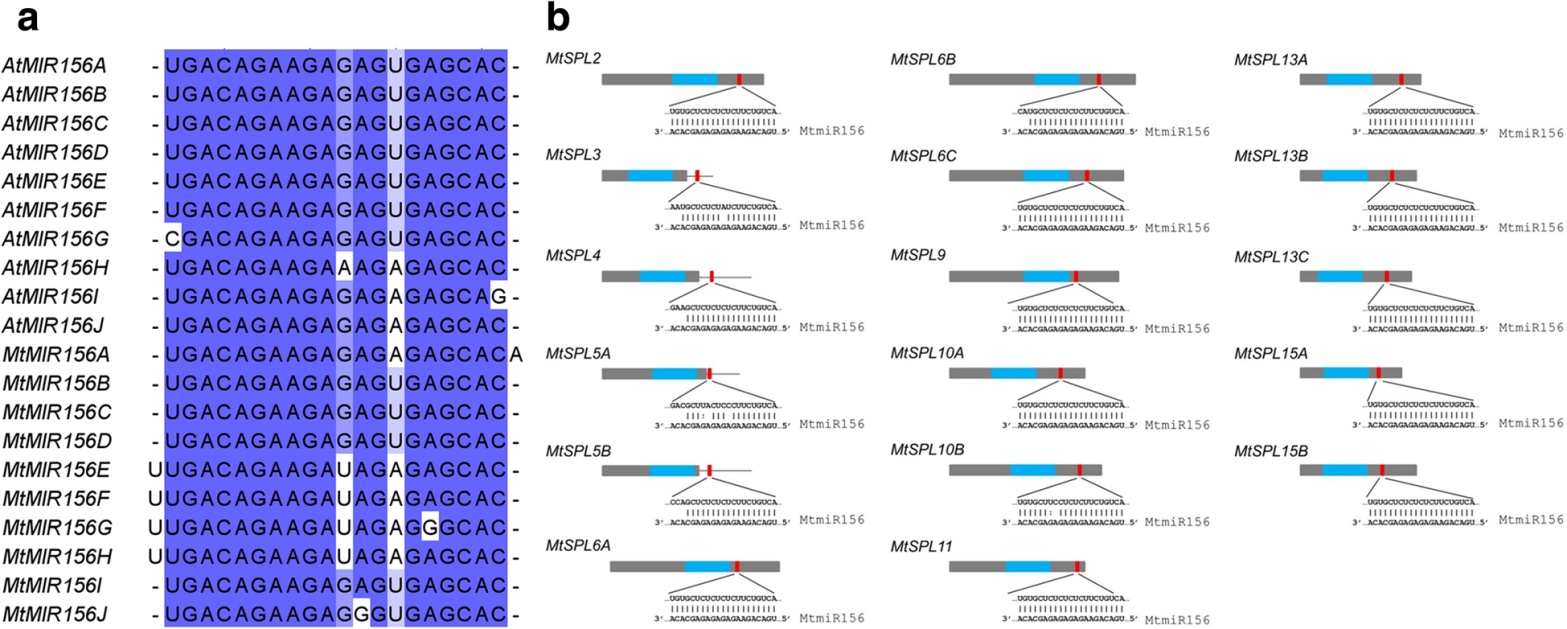
*miR156* sequences and binding sites of *MtmiR156* in *MtSPL* genes. **a** Mature *miR156* sequences in *A. thaliana* and *M. truncatula*. Shaded zones indicate the conserved sequences. **b** The *MtSPLs* regulated by *MtmiR156*. The gray boxes represent the CDS of *MtSPL* genes. The gray lines represent 3′ UTR of the *MtSPL* genes. The light blue boxes represent the conserved SBP domain. The red lines represent the *MtmiR156* target site

In order to understand the function of *miR156* and *miR156*-targeted *MtSPL* genes, we searched the miRBase Database and found 10 *MtmiR156* genes, *MtmiR156A-MtmiR156J*, in *M. truncatula* genome (Fig. 4a, Additional file 3a). Based on the *MtmiR156* precursor sequences, the stem-loop structures of *MtmiR156* were found (Additional file 3a). Previous studies showed that *miR156* could bind to the coding or 3′ UTR region of the *SPL* genes and reduce gene activity. Then, we used the online psRNATarget tool to find *MtmiR156* complementary sequences in the *MtSPL* transcripts. By comparison of the *MtmiR156* mature sequences and the *MtSPL* transcript sequences, we found that total 17 *MtSPL* genes have the *MtmiR156* binding sites, 13 of which were located in coding regions and 4 in 3′ UTR regions, respectively (Fig. 4b, Additional file 3b).

### Expression profile of *MtSPL* genes in different organs

**Fig. 5:**
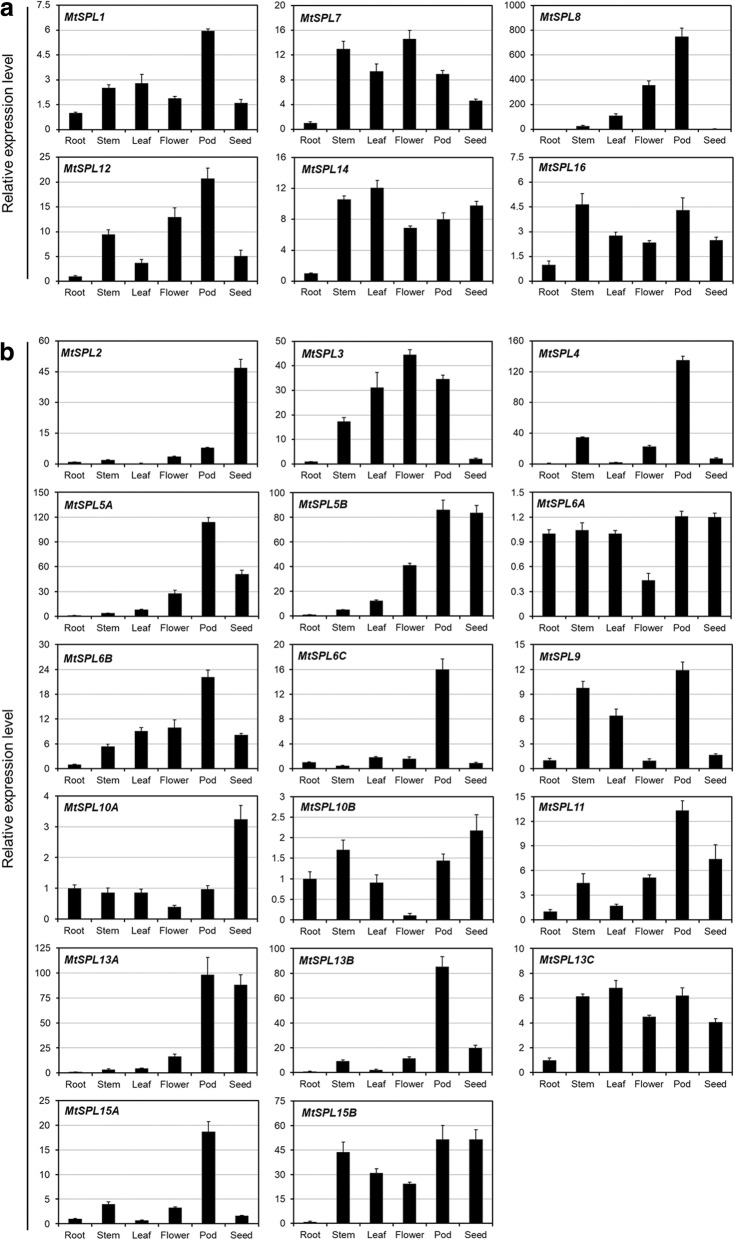
Expression patterns of *MtSPL* genes in six different organs. **a** Expression patterns of non-*MtmiR156*-targeted *MtSPL* genes. **b** Expression patterns of *MtmiR156*-targeted *MtSPL* genes

The expression pattern of a gene is often correlated with its function. In order to understand the developmental functions of the *MtSPL* genes, we investigated the expression profiles of the 23 *MtSPL* genes by quantitative real-time PCR (qRT-PCR) in six different organs, including roots, stems, leaves, flowers, pods, and seeds. qRT-PCR results showed that the relative expression levels and patterns of the *MtSPL* genes were varied in these organs (Fig. 5). For example, the non-*MtmiR156*-targeted *MtSPLs* (*MtSPL1*, *MtSPL7*, *MtSPL12*, *MtSPL14* and *MtSPL16*) were expressed in all of the organs tested, while *MtSPL8* was highly expressed in flower and pod (Fig. 5a). The *MtmiR156*-targeted *MtSPL* genes also showed differential expression profiles (Fig. 5b). Most *MtmiR156*-targeted *MtSPLs*, such as *MtSPL4*, *MtSPL5A*, *MtSPL5B*, *MtSPL6B*, *MtSPL6C*, *MtSPL11*, *MtSPL13B* and *MtSPL15A*, were highly detected in pod, implying their specific roles in pod development. *MtSPL2*, *MtSPL5B*, *MtSPL10A* and *MtSPL13A* were expressed at high levels in seed, indicating the possible involvement in seed development.

### *MtmiR156*-targeted *MtSPLs* play important roles in pod and seed development

**Fig. 6:**
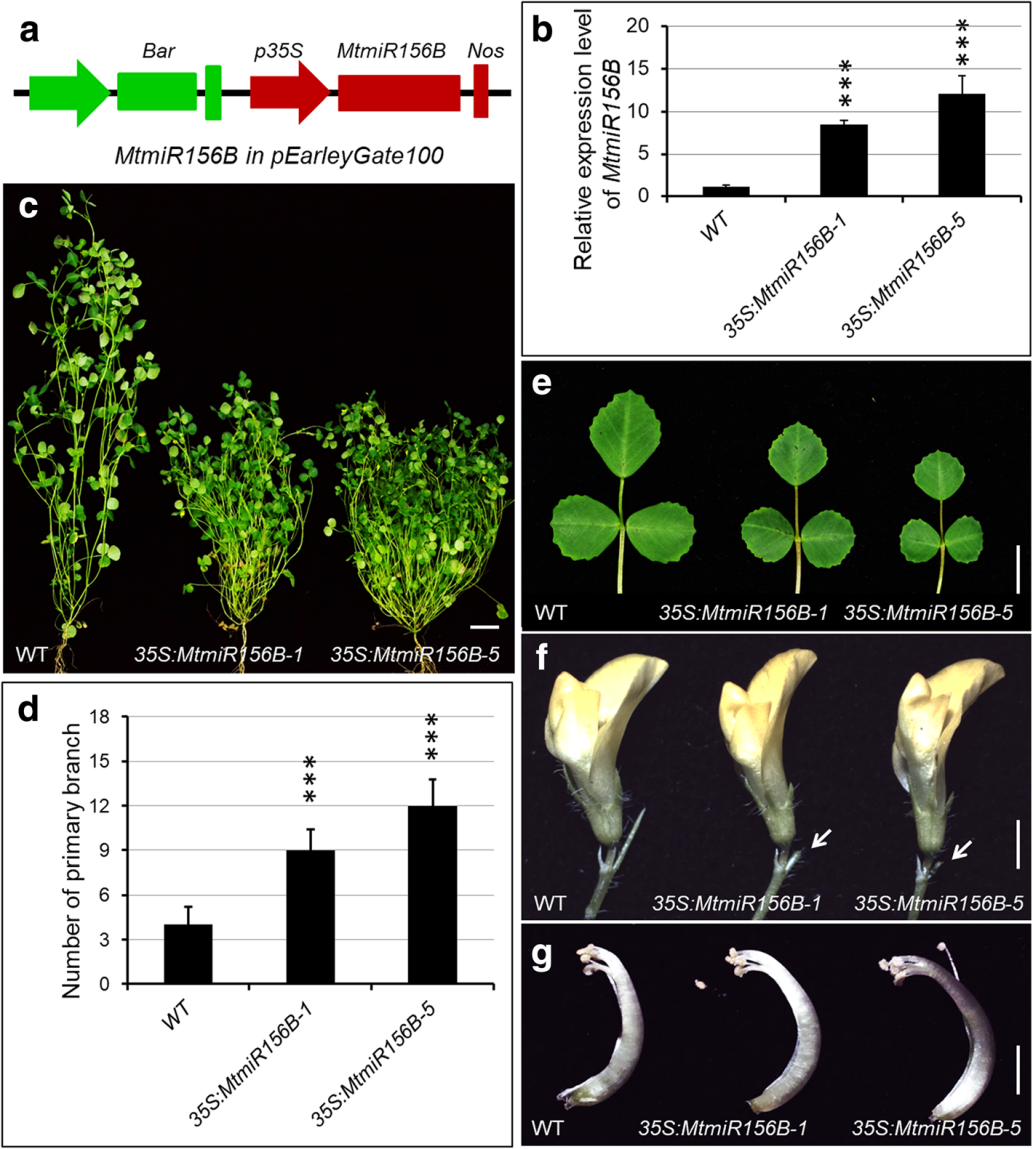
Developmental phenotypes of the *MtmiR156B*-overexpressing plants. **a** Schematic illustration of vector used for *MtmiR156B* overexpression. **b** Transcript levels of *MtmiR156B* in wild type and *35S:MtmiR156B* transgenic plants. **c** Two-month-old wild type and the *35S:MtmiR156B* transgenic plants. **d** The number of primary branch in the wild type and the *35S:MtmiR156B* transgenic plants. ^∗∗∗^*P <* 0.001. **e** Adult leaves of the wild type and *35S:MtmiR156B* transgenic plants. **f** Flower phenotype in the wild type and *35S:MtmiR156B* transgenic plants. Arrowheads indicated the developmental defects of spike in *MtmiR156B*-overexpressing plants. **g** The side view of the central carpel of wild type and *35S:MtmiR156B* transgenic plants. Bars: 5 cm (**c**), 1 cm (**e**), 2 mm (**f**), 2 mm (**g**)

To investigate the possible roles of the *MtmiR156*-targeted *MtSPLs* in growth and development of *M. truncatula*, the genomic sequence of *MtmiR156B* was cloned and introduced into wild type plants under the regulation of the cauliflower mosaic virus 35S promoter (Fig. 6a). Seven positive transgenic lines were obtained based on the PCR results (Additional file 4). The *MtmiR156B* was highly expressed in two transgenic plants (Fig. 6b). The *MtmiR156B*-overexpressing plants exhibited small leaves, increased lateral branches and reduced plant height, indicating that *MtmiR156B*-targeted *MtSPLs* play important roles in leaf morphogenesis, branching and stem elongation (Fig. 6c-e). Moreover, reduced function of the *MtmiR156B*-targeted *MtSPLs* also led to the defects in reproductive development. Compared with wild type, the spikes of the flowers in *MtmiR156B*-overexpressing plants were absent (Fig. 6f), however, the development of stamen and carpel was normal (Fig. 6g).

**Fig. 7:**
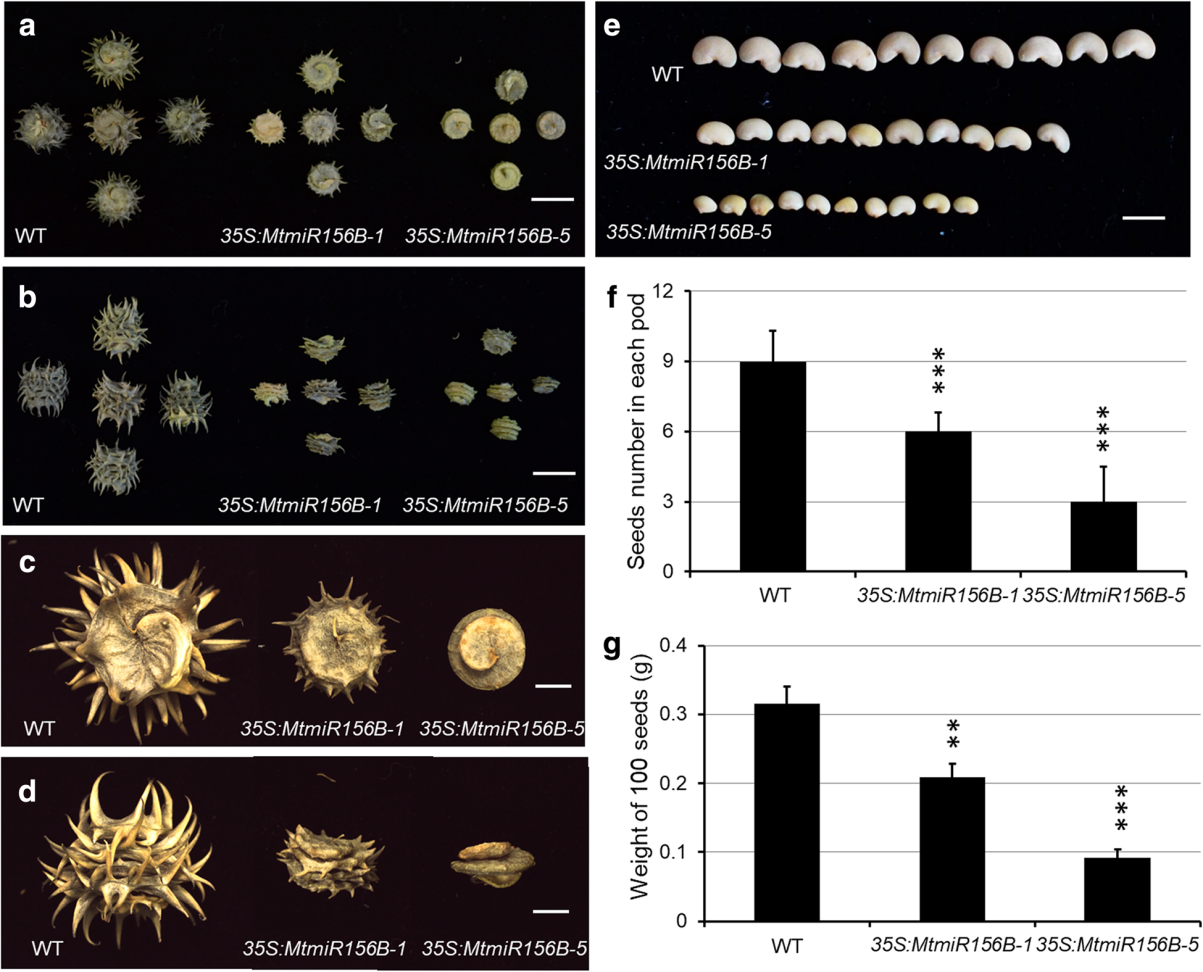
*MtmiR156*-targeted *MtSPL* genes are involved in pod and seed development. **a** and **b** The vertical (**a**) and side (**b**) view of pods of wild type and *35S:MtmiR156B* transgenic plants. **c** and **d** Close view of the pods in (**a**) and **(b)**. **e** Seeds of wild type and *35S:MtmiR156B* transgenic plants. **f** Seeds number in each pod in wild type and *35S:MtmiR156B* transgenic plants. **g** Weight of 100 seeds of wild type and *35S:MtmiR156B* transgenic plants. ^∗∗^*P <* 0.01, ^∗∗∗^*P <* 0.001. Bars: 1 cm (**a**, **b**), 2 mm (**c**, **d**), 5 mm (**e**)

Most *MtmiR156*-targeted *MtSPLs* were highly expressed in pod, implying that they may play redundant roles in pod development. Compared with the long slender spines developed on the pod surface in wild type, overexpression of *MtmiR156B* led to the reduction in pod size and the loss of spines on pod surface (Fig. 7a-d). The seed number in each pod, seed size and weight were significantly reduced in the *MtmiR156B-*overexpressing plants, compared with those in wild type (Fig. 7e-g). These results demonstrate that the *MtmiR156/MtSPL* regulation module is critical for the pod and seed development.

**Fig. 8:**
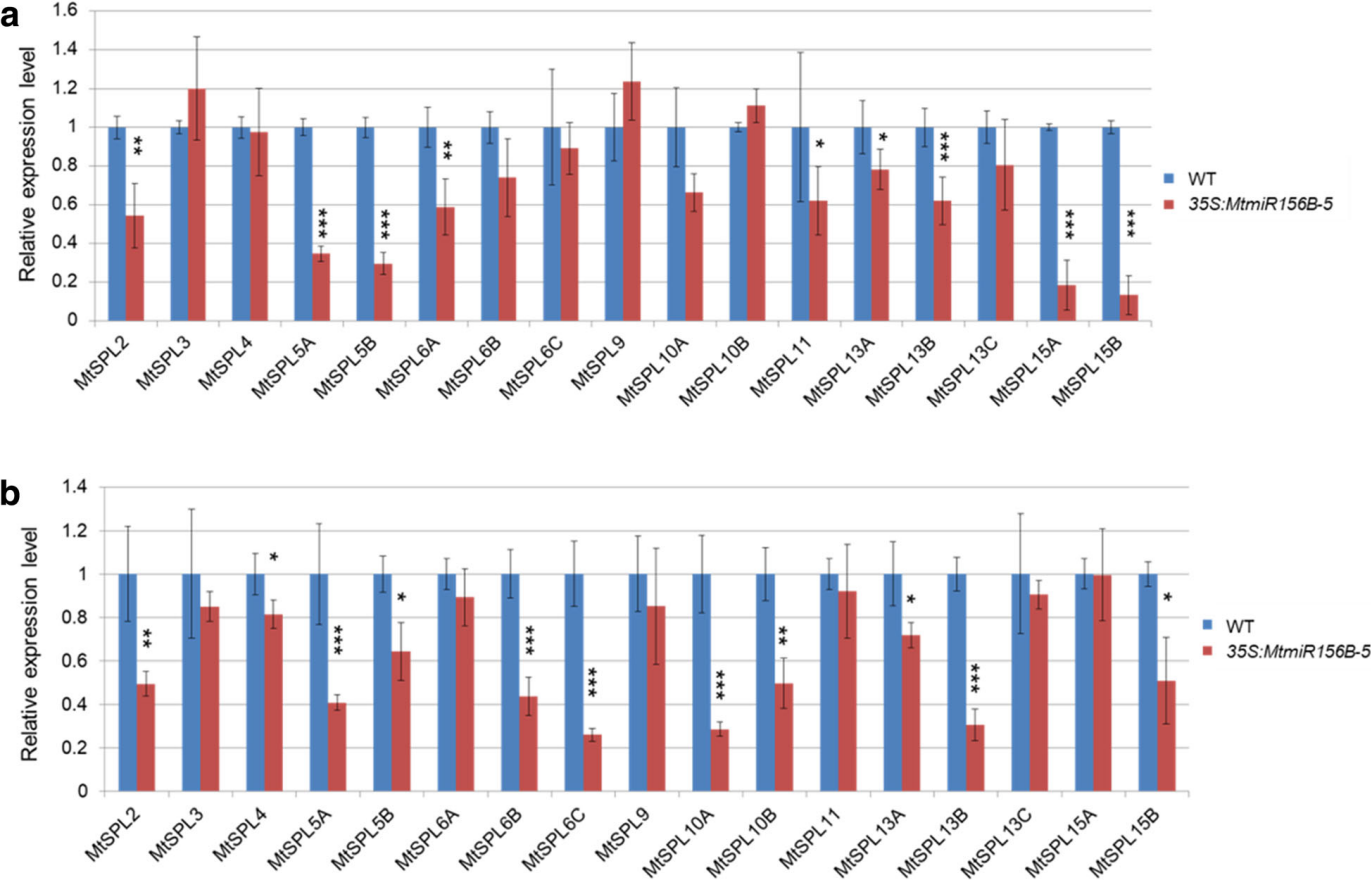
Expression levels of *MtmiR156*-targeted *SPL* genes in pod wall and seed of the wild type and *35S:MtmiR156B*-overexpressing plants. **a** Expression levels of the *MtmiR156*-targeted *SPLs* in pod wall. **b** Expression levels of the *MtmiR156*-targeted *SPLs* in seed. ^∗^*P <* 0.05, ^∗∗^*P <* 0.01, ^∗∗∗^*P <* 0.001

To further determine which *MtSPL* genes are involved in pod wall and seed development, the expression levels of all the *MtmiR156*-targeted *SPL* genes were analyzed in the pod wall and seed of the wild type and *MtmiR156B*-overexpressing plants. qRT-PCR results demonstrated that the expression of nine *MtSPLs* was significantly reduced in the pod wall of the *MtmiR156B*-overexpressing plants (Fig. 8a). The expression of *MtSPL5A*, *MtSPL5B*, *MtSPL15A*, and *MtSPL15B* showed over two-fold decrease in the transgenic plants, indicating that those *MtSPL* genes play crucial roles in spiky pod wall development (Fig. 8a). While, eleven *MtSPLs*, especially *MtSPL5A*, *MtSPL6B*, *MtSPL6C*, *MtSPL10A* and *MtSPL13B*, were downregulated in seed of the *MtmiR156B*-overexpressing plants, indicating the functional redundancy among those *MtSPL* genes during seed development (Fig. 8b).

## Discussion

Transcription factors play important roles during the processes of plant growth and development. The *SPL* genes encode a family of plant-specific transcription factors that contain the conserved SBP domain [2, 43]. In this study, through a genome-wide identification, we obtained 23 *MtSPL* genes from *M. truncatula* genome. Phylogenetic analysis showed that the *MtSPL* genes were more closely related to *Arabidopsis* than rice *SPL* genes, indicating that eudicots *SPL* genes may diverge from a common ancestor [44]. However, the number of *MtSPL* genes in *M. truncatula* is greater than that in *Arabidopsis* and rice, which contain 16 and 19 *SPLs*, respectively. Sequence homologous analysis suggested that faster gene duplication rates or species-specific gene duplication manners might play important roles in *SPL* gene expansion in *M. truncatula*. Gene structure and motif composition analyses showed that *MtSPL* genes within the same group shared similar motifs and exon/intron organization, suggesting that the functional evolution of *SPL* genes may be tightly correlated with the diversification of gene structure and conservation of motifs [6, 45].

Some *SPL* genes were posttranscriptionally regulated by *miR156* and involved in multiple developmental processes [4, 9, 11, 32, 36, 39–41, 45–49]. Based on the miRNA database information, ten *MtmiR156* genes were found in *M. truncatula* genome. The mature sequences of the *miR156* genes between *A. thaliana* and *M. truncatula* are similar, indicating the functional conservation of the *miR156* in different plant species [29]. It has been reported that 11 of 16 *SPLs* in *Arabidopsis* and 11 of 19 *SPLs* in rice contained the putative *miR156* binding sites [2, 7, 11, 12, 17, 36, 46]. By searching the *MtmiR156* complementary sequence in *MtSPLs* mRNAs, we found that 17 *MtSPLs* out of 23 contained the putative *MtmiR156* binding sites, suggesting the conservation of *miR156*-mediated posttranscriptional regulation in plants.

In *Arabidopsis*, loss-of-function of multiple *SPL* genes or overexpression of *AtmiR156A* resulted in the decreased rosette leaf area [46]. In tomato and petunia, overexpressing *AtMIR156B* or *PhSBP1*code-silenced plants produced the higher number of small leaves and branches [47, 50]. Furthermore, overexpression of *OsmiR156B* in switchgrass and loss-of-function in *IPA1* in rice and down-regulation of *MsSPL13* in alfalfa also led to the increased number of branches [39, 40, 51, 52]. In accordance with these reports, the similar phenotypes, including more branches and small leaves, were also displayed in *MtmiR156B*-overexpressing plants. These observations indicate that *MtmiR156/MtSPL* regulation module plays the conserved roles in vegetative development.

The seeds of legume are developed within an ovary-derived pod, whose walls provide numerous functions, such as the protection of the seeds and the production of photosynthates [53–55]. Seed dispersal is the transport of pods/seeds away from the parent plant and has been implicated in rapid plant migration and the spread of invasive [56–59]. The pods in many plant species develop the spines or stiff hairs, such as *Trifolium angustifolium*, *M. polymorpha* and *M. truncatula* [60, 61]. Such structures are very important for seed migration, because seed pods can adhere to animals by means of spines or hairs, and be transported on the outside [60, 62]. So, the proper development of spines/hairs on pod is critical for seed dispersal, along with species diversity or ecological invasion. The pod and pod wall of *M. truncatula* are helical and thick with spines [63–65]. In this study, we found that most *MtmiR156*-targeted *MtSPL* genes were highly expressed in pod and seed. Moreover, seed size and number in *MtmiR156B*-overexpressing plants were decreased. Importantly, the development of spines on pod was also defective, due to the downregulation of several *MtmiR156*-targeted *MtSPLs*. These observations indicate that the *MtmiR156/MtSPL* regulation module may contribute to the genetic variability through the regulation of pod morphology.

In *Arabidopsis*, ectopic expression of the *TaSPL16* results in early flowering and increase of organ size and yield [66]. This finding implies that *SPL* is possible for the improvement of seed production in legume species. In this study, *35S:MtmiR156B* plants show defective spines on pod in *M. trunctula*. However, the number of lateral branches is increased dramatically. The biomass is a critical trait in evaluation of forage grass. Therefore, downregulation of targeted *SPL* genes by overexpression of *miR156* in legume forage, such as alfalfa, may provide a helpful tool to improve forage production.

## Conclusion

In this study, we performed genome-wide analyses and identified *SPL* genes in *M. truncatula*. The genetic redundancy of *MtSPL* genes hinders the discoveries of their potential functions. However, the phenotypes of *MtmiR156B*-overexpressing plants reveal that *MtmiR156/MtSPL* modules are not only involved in the development of leaves and branches, but also indirectly contribute to seed dispersal by controlling the formation of spine on pods. Characterization of the loss-of-function *MtSPL* mutants may help to provide insight into the roles of *MtmiR156/MtSPL* module in the development of spine of pod, and shed light on the new function of SPL family among plant species.

## Methods

### Plant materials and growth conditions

*Medicago truncatula* R108 ecotype was used as the wild type, which is obtained from the Noble Research Institution, USA. The seeds of *35S:MtmiR156B* and wild type were scarified with sandpaper and treated at 4 °C for 5 days. The geminated seeds were transferred to nursery seedling plate (4 × 4 × 5 cm Length, Width, Height) for 2 weeks. Then, the seedlings were transferred to Luqing soil mix (soil: vermiculite = 3:1) and grown in the greenhouse at 22 °C (day) / 22 °C (night) with 16 h (day) / 8 h (night) photoperiod, and relative humidity at 70 to 80%.

### Identification and phylogenetic analysis of *SPL* genes in *M. truncatula*

To identify the *SPL* genes in *M. truncatula* genome, firstly, we used 16 AtSPL and 19 OsSPL protein sequences to execute BLASTP search the *Medicago truncatula* Genome Database (http://www.medicagogenome.org/). The AtSPL protein sequences were obtained from The Arabidopsis Information Resource (TAIR, http://www.arabidopsis.org/). The OsSPL protein sequences were obtained from the Rice Genome Annotation Project (http://rice.plantbiology.msu.edu/). Totally, 24 putative *MtSPL* genes were identified in *M. truncatula* genome using blast with a cut-off E-value >1e^− 3^. Secondly, we searched the Plant Transcription Factor Database (http://planttfdb.cbi.pku.edu.cn/) and confirmed the blast search result. Thirdly, the 24 MtSPL protein sequences were further analyzed on the NCBI Conserved Domain Search website (https://www.ncbi.nlm.nih.gov/Structure/cdd/cdd.shtml) and found that Medtr8g066240 lost the conserved SBP domain. Therefore, Medtr8g066240 was excluded from the putative *MtSPL* genes, and total 23 *MtSPL* genes were used for study.

Multiple protein sequence alignment was performed using Jalview software (http://www.jalview.org/). The phylogenetic tree for *Arabidopsis*, rice, and *Medicago SPL* gene family members was constructed using MEGA 7.0.

### Chromosome location and gene structure of *MtSPL* genes

The informations of *MtSPL* genes on chromosome location, including chromosome length, gene direction, and gene start and stop position, were obtained from the *M. truncatula* genome database. Exon / intron structures of *MtSPL* genes were determined by aligning the coding sequences and their corresponding genomic sequences using the online Gene Structure Display Server (GSDS, http://gsds.cbi.pku.edu.cn/) website.

### The identification of conserved domain and the prediction of *MtmiR156*-targeted *MtSPLs* prediction

The online MEME tool (http://meme-suite.org/) was used to predict both conserved domains and potential motifs in the 23 MtSPL proteins with the following parameters: maximum number of motifs to find, 20; minimum width of motif, 6; maximum width of motif, 100; minimum number of sites for each motif, 2. The mature sequences of *M. truncatula MtmiR156A* to *MtmiR156J* were obtained from miRBase database (http://www.mirbase.org/). The *MtmiR156*-targeted *MtSPL* genes and their binding sites were obtained by searching the gene coding and UTR regions on the psRNATarget (http://plantgrn.noble.org/psRNATarget/home) website.

### RNA extraction and gene expression analysis

The samples in 60-day old wild-type plants were used for RNA extraction. For gene expression pattern analysis, the roots, leaves, flowers, pods and seeds samples were harvested from primary roots, fully expanded leaves, fully opened flowers, 20-day post-pollination pods and seeds. To analyze the relative expression levels of *MtmiR156*-targeted *MtSPL* genes in the *MtmiR156B*-overexpressing plants, 20-day post-pollination seeds and pod walls were collected from wild type and *35S:MtmiR156B* transgenic plants. To analyze the relative expression levels of *MtmiR156B* in the *MtmiR156B*-overexpressing plants, 60-day old fully expanded leaves were collected from wild type and *35S:MtmiR156B* transgenic plants.

Total RNA of these organs was extracted using the Trizol-RT Reagent (Molecular Research Center, INC) following the manufacturer’s instructions. The quality and quantity of the extracted RNA were analyzed using Nanodrop 2000 Spectrophotometer (Thermo Scientific, USA). Reverse transcription PCR was performed with 2.5 μg total RNA using Roche First Strand cDNA Synthesis Kit (Roche, USA). Then, the cDNA was diluted to 20 ng/μl with DEPC treated H_2_O. For quantitative real-time PCR (qRT-PCR) analysis, the primers of the 23 *MtSPL* genes were designed by Primer Express 3.0 software (Additional file 5). qRT-PCR was executed in triplicate for each organ on CFX Connect™ (Bio-Rad, USA) using FastStart Essential DNA Green Master Kit (Roche, USA). The *MtUBI* gene was selected as internal control, and the relative expression levels of different *MtSPL* genes were calculated using 2^-△△CT^ method [67].

### Plasmid construction and plant transformation

To obtain the *MtmiR156B* overexpression construction, the 830 bp DNA sequence containing the *MtmiR156B* stem-loop structure was amplified using primer pair MtmiR156B-F/MtmiR156B-R. The *MtmiR156B* sequence was transferred into the pEarleyGate 100 vector by Gateway LR reaction (Invitrogen, USA) [68]. Then *35S:MtmiR156B* destination construct was introduced into *Agrobacterium* strain EHA105. For stable transformation, leaves of wild type were transformed with EHA105 strain containing the *35S:MtmiR156B* vector [69].

## Additional files

Additional file 1: The sequences of all genes involved in this study. (DOCX 31 kb)

Additional file 2: Multiple amino acid sequences alignment of MtSPL proteins using full-length amino acid sequences. Sequences were aligned using Jalview software. (DOCX 575 kb)

Additional file 3: Secondary structure of *MtmiR156* and regulation of *MtSPLs* by *MtmiR156*. ^a^ RNA secondary structures of the *MtmiR156A-MtmiR156A J* were predicted by the online mfold Web Server. The nucleotides with light green color in stem-loop structures indicate the mature *MtmiR156* sequences. ^b^ Multiple *MtSPL* genes were regulated by *MtmiR156*. The deoxyribonucleotide with shaded color indicates the conserved sequences targeted by *MtmiR156*. (DOCX 548 kb)

Additional file 4: Molecular characterization of *MtmiR156B* overexpression lines. PCR analysis was performed using primer pair 35S-F/MtmiR156B-R for regenerated transgenic plants together with the positive control (*35S:MtmiR156B*), and negative control (Wild-type). (DOCX 168 kb)

Additional file 5: Primers used in this study. (DOCX 23 kb)

## Data Availability

Accession numbers: MtSPL1: Medtr1g086250; MtSPL2: Medtr3g085180; MtSPL3: Medtr4g088555; MtSPL4:Medtr2g014200; MtSPL5A:Medtr2g078770; MtSPL5B:Medtr8g463140; MtSPL6A:Medtr5g046670; MtSPL6B:Medtr2g461920; MtSPL6C:Medtr4g109770; MtSPL7:Medtr2g020620; MtSPL8:Medtr8g005960; MtSPL9:Medtr7g444860; MtSPL10A:Medtr8g080680; MtSPL10B:Medtr8g080670; MtSPL11:Medtr8g080690; MtSPL12:Medtr7g110320; MtSPL13A:Medtr8g096780; MtSPL13B:Medtr3g099080; MtSPL13C:Medtr7g028740; MtSPL14:Medtr1g035010; MtSPL15A:Medtr7g092930; MtSPL15B:Medtr1g053715; MtSPL16:Medtr2g046550; MtmiR156B: MIMAT0011057. The sequences of all genes involved in this study are listed in Additional file 1, and also can be found in NCBI database. The plant materials in this study are available from the corresponding author on request.

## Abbreviations

*AP1*: *APETALA1*
*FT*: *FLOWERING LOCUS T*
*FUL*: *FRUITFULL*
GA: Gibberellin
*LFY*: *LEAFY*
NJ: Neighbor-Joining
NLS: Nuclear localization signal
qRT-PCR: Quantitative real-time PCR
SBP: SQUAMOSA PROMOTER BINDING PROTEIN
*SOC1*: *SUPPRESSOR OF OVEREXPRESSION OF CO 1*
*SPL*: *SQUAMOSA PROMOTER BINDING PROTEIN-LIKE*
TFs: Transcription factors
UTR: Untranslated region
Zn1: Zinc finger 1
Zn2: Zinc finger 2
