# Supplementary material for: Genome-wide characterization of SPL family in Medicago truncatula reveals the novel roles of miR156/SPL module in spiky pod development
Source: BMC Genomics. 2019 Jul 5;20:552. doi: 10.1186/s12864-019-5937-1 (PMC6612136; doi:10.1186/s12864-019-5937-1)
Supplement: Supplementary file 1 — The sequences of all genes involved in this study. (DOCX 31 kb) [file 12864_2019_5937_MOESM1_ESM.docx]

>AtSPL1

MEARIDEGGEAQQFYGSVGKRSVEWDLNDWKWDGDLFLATQTTRGRQFFPLGNSSNSSSSCSDEGNDKKRRAVAIQGDTNGALTLNLNGESDGLFPAKKTKSGAVCQVENCEADLSKVKDYHRRHKVCEMHSKATSATVGGILQRFCQQCSRFHLLQEFDEGKRSCRRRLAGHNKRRRKTNPEPGANGNPSDDHSSNYLLITLLKILSNMHNHTGDQDLMSHLLKSLVSHAGEQLGKNLVELLLQGGGSQGSLNIGNSALLGIEQAPQEELKQFSARQDGTATENRSEKQVKMNDFDLNDIYIDSDDTDVERSPPPTNPATSSLDYPSWIHQSSPPQTSRNSDSASDQSPSSSSEDAQMRTGRIVFKLFGKEPNEFPIVLRGQILDWLSHSPTDMESYIRPGCIVLTIYLRQAETAWEELSDDLGFSLGKLLDLSDDPLWTTGWIYVRVQNQLAFVYNGQVVVDTSLSLKSRDYSHIISVKPLAIAATEKAQFTVKGMNLRQRGTRLLCSVEGKYLIQETTHDSTTREDDDFKDNSEIVECVNFSCDMPILSGRGFMEIEDQGLSSSFFPFLVVEDDDVCSEIRILETTLEFTGTDSAKQAMDFIHEIGWLLHRSKLGESDPNPGVFPLIRFQWLIEFSMDREWCAVIRKLLNMFFDGAVGEFSSSSNATLSELCLLHRAVRKNSKPMVEMLLRYIPKQQRNSLFRPDAAGPAGLTPLHIAAGKDGSEDVLDALTEDPAMVGIEAWKTCRDSTGFTPEDYARLRGHFSYIHLIQRKINKKSTTEDHVVVNIPVSFSDREQKEPKSGPMASALEITQIPCKLCDHKLVYGTTRRSVAYRPAMLSMVAIAAVCVCVALLFKSCPEVLYVFQPFRWELLDYGTS

>AtSPL2

MECNAKPPFQWELENLISFGTSTAEVPRKLKPMEWEIDGFDCTSLYSSSFAYAGSSGSDIAHAFSKSSKSTSISSSSAEVRTHNFTSETGESLPGEFAKGIDTSPSLELSFGSGDPVLGLKLGKRTYFEDFWEVENAKGLGLPVTLASSSVSPVKKSKSIPQRLQTPHCQVEGCNLDLSSAKDYHRKHRICENHSKFPKVVVSGVERRFCQQCSRFHCLSEFDEKKRSCRRRLSDHNARRRKPNPGRTYDGKPQVDFVWNRFALIHPRSEEKFIWPSSKHVPSRVLMPQPAKTEISDTEHNRFGLLDPKTKTARAELFSKEKVTISSHMGASQDLDGALSLLSNSTTWVSSSDQPRRFTLDHHPSSNLQPVAHRSAAQLNSVSGYWQPDPPAVEGPTALHRNGVGQFNENYFSLNQFYN

>AtSPL3

MSMRRSKAEGKRSLRELSEEEEEEEETEDEDTFEEEEALEKKQKGKATSSSGVCQVESCTADMSKAKQYHKRHKVCQFHAKAPHVRISGLHQRFCQQCSRFHALSEFDEAKRSCRRRLAGHNERRRKSTTD

>AtSPL4

MEGKRSQGQGYMKKKSYLVEEDMETDTDEEEEVGRDRVRGSRGSINRGGSLRLCQVDRCTADMKEAKLYHRRHKVCEVHAKASSVFLSGLNQRFCQQCSRFHDLQEFDEAKRSCRRRLAGHNERRRKSSGESTYGEGSGRRGINGQVVMQNQERSRVEMTLPMPNSSFKRPQIR

>AtSPL5

MEGQRTQRRGYLKDKATVSNLVEEEMENGMDGEEEDGGDEDKRKKVMERVRGPSTDRVPSRLCQVDRCTVNLTEAKQYYRRHRVCEVHAKASAATVAGVRQRFCQQCSRFHELPEFDEAKRSCRRRLAGHNERRRKISGDSFGEGSGRRGFSGQLIQTQERNRVDRKLPMTNSSFKRPQIR

>AtSPL6

MDSWSYGRSVFMSNETLLPCDTFAKNRRFEQRLSNNDDVLISDMAGNSNGFSAVSITKVVPEEEDEENISSSSKFSSQELNRIDFKLRSFLDLGNDDDDTSSRGFALPSKKSRASNLCSQNPLCQVYGCSKDLSSSKDYHKRHRVCEAHSKTSVVIVNGLEQRFCQQCSRFHFLSEFDDGKRSCRRRLAGHNERRRKPAFYFLPGKRHKLLRTSQDVVGNKFLENSSLVLPESFPGSLLYRVIDEDDHRTSRLVSFKDEPTCSMFPTNEQNSSRTYESKPAIYSTEVSSIWDLHETAASRSTRALSLLSAQSQQHLSKFPNTTFSITQPNQNLNHSSSTDYHQMEQPLWIDPGKTNSAGSSSCKGKGTSTVDLLQLSSHLQRIEQQRNYTGDVKQEYNELYFPGS

>AtSPL7

MSSLSQSPPPPEMDIQPPALVNDDPSTYSSALWDWGDLLDFAADERLLVDQIHFPPVLSPPLPPLIPTQTPAESELDPSPEESGSGSDRVRKRDPRLICSNFIEGMLPCSCPELDQKLEDAELPKKKRVRGGSGVARCQVPDCEADISELKGYHKRHRVCLRCATASFVVLDGENKRYCQQCGKFHLLPDFDEGKRSCRRKLERHNNRRKRKPVDKGGVAAEQQQVLSQNDNSVIDVEDGKDITCSSDQRAEEEPSLIFEDRHITTQGSVPFTRSINADNFVSVTGSGEAQPDEGMNDTKFERSPSNGDNKSAYSTVCPTGRISFKLYDWNPAEFPRRLRHQIFQWLANMPVELEGYIRPGCTILTVFIAMPEIMWAKLSKDPVAYLDEFILKPGKMLFGRGSMTVYLNNMIFRLIKGGTTLKRVDVKLESPKLQFVYPTCFEAGKPIELVVCGQNLLQPKCRFLVSFSGKYLPHNYSVVPAPDQDGKRSCNNKFYKINIVNSDPSLFGPAFVEVENESGLSNFIPLIIGDAAVCSEMKLIEQKFNATLFPEGQEVTACSSLTCCCRDFGERQSTFSGLLLDIAWSVKVPSAERTEQPVNRCQIKRYNRVLNYLIQNNSASILGNVLHNLETLVKKMEPDSLVHCTCDCDVRLLHENMDLASDIHRKHQSPIESKVNPPSSGCCCVSSQKDIPSRILNFNKDPEAGLDCKERIQADCSPDSGGKETDPLLNKEVVMNVNDIGDWPRKSCIKTHSALAFRSRQTMFLIATFAVCFAVCAVLYHPNKVTQLAVAIRMRLVHKI

>AtSPL8

MLDYEWDNPSSIVLSGDERNPDSDPTRSSFSFFDPISHYNNDHRHITISPPLLSSFSNQQQQHHLTLYGQTNSNNQFLHHHHHHHSLYGSTTTTTPYGASDPIYHPHSSAPPASLFSYDQTGPGSGSGSSYNFLIPKTEVDFTSNRIGLNLGGRTYFSAADDDFVSRLYRRSRPGESGMANSLSTPRCQAEGCNADLSHAKHYHRRHKVCEFHSKASTVVAAGLSQRFCQQCSRFHLLSEFDNGKRSCRKRLADHNRRRRKCHQSASATQDTGTGKTTPKSPNDSGVKASSSPSSNAPPTISLECFRQRQFQTTASSSTSASSSSNSMFFSSG

>AtSPL9

MEMGSNSGPGHGPGQAESGGSSTESSSFSGGLMFGQKIYFEDGGGGSGSSSSGGRSNRRVRGGGSGQSGQIPRCQVEGCGMDLTNAKGYYSRHRVCGVHSKTPKVTVAGIEQRFCQQCSRFHQLPEFDLEKRSCRRRLAGHNERRRKPQPASLSVLASRYGRIAPSLYENGDAGMNGSFLGNQEIGWPSSRTLDTRVMRRPVSSPSWQINPMNVFSQGSVGGGGTSFSSPEIMDTKLESYKGIGDSNCALSLLSNPHQPHDNNNNNNNNNNNNNNTWRASSGFGPMTVTMAQPPPAPSQHQYLNPPWVFKDNDNDMSPVLNLGRYTEPDNCQISSGTAMGEFELSDHHHQSRRQYMEDENTRAYDSSSHHTNWSL

>AtSPL10

MDCNMVSSFPWDWENLIMSNQSKTENEKKQQSTEWEFEKGEGIESIVPDFLGFEKVSSGSATSFWHTAVSKSSQSTSINSSSPEDKRCNLASQSSPGDSSSNIDFLQVKPSTALEVPIASAESDLCLKLGKRTYSEEFWGRNNNDLSAVSMNLLTPSVVARKKTKSCGQSMQVPRCQIDGCELDLSSSKDYHRKHRVCETHSKCPKVVVSGLERRFCQQCSRFHAVSEFDEKKRSCRKRLSHHNARRRKPQGVFPLNSERVFDRRQHTSMLWNGLSLNTRSEEKYTWGTTYETKPTQMESGFTLSFQRGNGSEDQLFTGSTLSFSAFQTSGGFSAGKSNIQLPDKGVGECSGGLHESHDFYSALSLLSTTSDSQGIKHTPVAEPPPIFGTFPSHFI

>AtSPL11

MDCNMVSSSQWDWEHLIMSNPSRTEDDSKQLPTEWEIEKGEGIESIVPHFSGLERVSSGSATSFWHTAVSKSSQSTSINSSSPEAKRCKLASESSPGDSCSNIDFVQVKAPTALEVSVASAESDLCLKLGKRTYSEEYWGRNNNEISAVSMKLLTPSVVAGKSKLCGQSMPVPRCQIDGCELDLSSAKGYHRKHKVCEKHSKCPKVSVSGLERRFCQQCSRFHAVSEFDEKKRSCRKRLSHHNARRRKPQGVFSMNPERVYDRRQHTNMLWNGVSLNARSEEMYEWGNNTYDTKPRQTEKSFTLSFQRGNGSEDQLVASSSRMFSTSQTSGGFPAGKSKFQLHGEDVGEYSGVLHESQDIHRALSLLSTSSDPLAQPHVQPFSLLCSYDVVPK

>AtSPL12

MEARIEGEVEGHSLEYGFSGKRSVEWDLNDWKWNGDLFVATQLNHGSSNSSSTCSDEGNVEIMERRRIEMEKKKKRRAVTVVAMEEDNLKDDDAHRLTLNLGGNNIEGNGVKKTKLGGGIPSRAICCQVDNCGADLSKVKDYHRRHKVCEIHSKATTALVGGIMQRFCQQCSRFHVLEEFDEGKRSCRRRLAGHNKRRRKANPDTIGNGTSMSDDQTSNYMLITLLKILSNIHSNQSDQTGDQDLLSHLLKSLVSQAGEHIGRNLVGLLQGGGGLQASQNIGNLSALLSLEQAPREDIKHHSVSETPWQEVYANSAQERVAPDRSEKQVKVNDFDLNDIYIDSDDTTDIERSSPPPTNPATSSLDYHQDSRQSSPPQTSRRNSDSASDQSPSSSSGDAQSRTDRIVFKLFGKEPNDFPVALRGQILNWLAHTPTDMESYIRPGCIVLTIYLRQDEASWEELCCDLSFSLRRLLDLSDDPLWTDGWLYLRVQNQLAFAFNGQVVLDTSLPLRSHDYSQIITVRPLAVTKKAQFTVKGINLRRPGTRLLCTVEGTHLVQEATQGGMEERDDLKENNEIDFVNFSCEMPIASGRGFMEIEDQGGLSSSFFPFIVSEDEDICSEIRRLESTLEFTGTDSAMQAMDFIHEIGWLLHRSELKSRLAASDHNPEDLFSLIRFKFLIEFSMDREWCCVMKKLLNILFEEGTVDPSPDAALSELCLLHRAVRKNSKPMVEMLLRFSPKKKNQTLAGLFRPDAAGPGGLTPLHIAAGKDGSEDVLDALTEDPGMTGIQAWKNSRDNTGFTPEDYARLRGHFSYIHLVQRKLSRKPIAKEHVVVNIPESFNIEHKQEKRSPMDSSSLEITQINQCKLCDHKRVFVTTHHKSVAYRPAMLSMVAIAAVCVCVALLFKSCPEVLYVFQPFRWELLEYGTS

>AtSPL13

MDWNFKLSSGYLSGFDQEPDLSPMDGSISFGGSSQSKADFSFDLKLGRNIGNSSSVFGDTEQVISLSKWKDSALAKPEGSRSSSSKRTRGNGVGTNQMPICLVDGCDSDFSNCREYHKRHKVCDVHSKTPVVTINGHKQRFCQQCSRFHALEEFDEGKRSCRKRLDGHNRRRRKPQPEHIGRPANFFTGFQGSKLLEFSGGSHVFPTTSVLNPSWGNSLVSVAVAANGSSYGQSQSYVVGSSPAKTGIMFPISSSPNSTRSIAKQFPFLQEEESSRTASLCERMTSCIHDSDCALSLLSSSSSSVPHLLQPPLSLSQEAVETVFYGSGLFENASAVSDGSVISGNEAVRLPQTFPFHWE

>AtSPL14

MDEVGAQVAAPMFIHQSLGRKRDLYYPMSNRLVQSQPQRRDEWNSKMWDWDSRRFEAKPVDVEVQEFDLTLRNRSGEERGLDLNLGSGLTAVEETTTTTQNVRPNKKVRSGSPGGNYPMCQVDNCTEDLSHAKDYHRRHKVCEVHSKATKALVGKQMQRFCQQCSRFHLLSEFDEGKRSCRRRLAGHNRRRRKTTQPEEVASGVVVPGNHDTTNNTANANMDLMALLTALACAQGKNAVKPPVGSPAVPDREQLLQILNKINALPLPMDLVSKLNNIGSLARKNMDHPTVNPQNDMNGASPSTMDLLAVLSTTLGSSSPDALAILSQGGFGNKDSEKTKLSSYENGVTTNLEKRTFGFSSVGGERSSSSNQSPSQDSDSRGQDTRSSLSLQLFTSSPEDESRPTVASSRKYYSSASSNPVEDRSPSSSPVMQELFPLQASPETMRSKNHKNSSPRTGCLPLELFGASNRGAADPNFKGFGQQSGYASSGSDYSPPSLNSDAQDRTGKIVFKLLDKDPSQLPGTLRSEIYNWLSNIPSEMESYIRPGCVVLSVYVAMSPAAWEQLEQKLLQRLGVLLQNSPSDFWRNARFIVNTGRQLASHKNGKVRCSKSWRTWNSPELISVSPVAVVAGEETSLVVRGRSLTNDGISIRCTHMGSYMAMEVTRAVCRQTIFDELNVNSFKVQNVHPGFLGRCFIEVENGFRGDSFPLIIANASICKELNRLGEEFHPKSQDMTEEQAQSSNRGPTSREEVLCFLNELGWLFQKNQTSELREQSDFSLARFKFLLVCSVERDYCALIRTLLDMLVERNLVNDELNREALDMLAEIQLLNRAVKRKSTKMVELLIHYLVNPLTLSSSRKFVFLPNITGPGGITPLHLAACTSGSDDMIDLLTNDPQEIGLSSWNTLRDATGQTPYSYAAIRNNHNYNSLVARKLADKRNKQVSLNIEHEVVDQTGLSKRLSLEMNKSSSSCASCATVALKYQRRVSGSQRLFPTPIIHSMLAVATVCVCVCVFMHAFPIVRQGSHFSWGGLDYGSI

>AtSPL15

MELLMCSGQAESGGSSSTESSSLSGGLRFGQKIYFEDGSGSRSKNRVNTVRKSSTTARCQVEGCRMDLSNVKAYYSRHKVCCIHSKSSKVIVSGLHQRFCQQCSRFHQLSEFDLEKRSCRRRLACHNERRRKPQPTTALFTSHYSRIAPSLYGNPNAAMIKSVLGDPTAWSTARSVMQRPGPWQINPVRETHPHMNVLSHGSSSFTTCPEMINNNSTDSSCALSLLSNSYPIHQQQLQTPTNTWRPSSGFDSMISFSDKVTMAQPPPISTHQPPISTHQQYLSQTWEVIAGEKSNSHYMSPVSQISEPADFQISNGTTMGGFELYLHQQVLKQYMEPENTRAYDSSPQHFNWSL

>AtSPL16

MGELPKDDWQMNRWKWDGQRFEAIELQGESLQLSNKKGLDLNLPCGFNDVEGTPVDLTRPSKKVRSGSPGSGGGGGGNYPKCQVDNCKEDLSIAKDYHRRHKVCEVHSKATKALVGKQMQRFCQQCSRFHLLSEFDEGKRSCRRRLDGHNRRRRKTQPDAITSQVVALENRDNTSNNTNMDVMALLTALVCAQGRNEATTNGSPGVPQREQLLQILNKIKALPLPMNLTSKLNNIGILARKNPEQPSPMNPQNSMNGASSPSTMDLLAALSASLGSSAPEAIAFLSQGGFGNKESNDRTKLTSSDHSATTSLEKKTLEFPSFGGGERTSSTNHSPSQYSDSRGQDTRSSLSLQLFTSSPEEESRPKVASSTKYYSSASSNPVEDRSPSSSPVMQELFPLHTSPETRRYNNYKDTSTSPRTSCLPLELFGASNRGATANPNYNVLRHQSGYASSGSDYSPPSLNSNAQERTGKISFKLFEKDPSQLPNTLRTEIFRWLSSFPSDMESFIRPGCVILSVYVAMSASAWEQLEENLLQRVRSLVQDSEFWSNSRFLVNAGRQLASHKHGRIRLSKSWRTLNLPELITVSPLAVVAGEETALIVRGRNLTNDGMRLRCAHMGNYASMEVTGREHRLTKVDELNVSSFQVQSASSVSLGRCFIELENGLRGDNFPLIIANATICKELNRLEEEFHPKDVIEEQIQNLDRPRSREEVLCFLNELGWLFQRKWTSDIHGEPDFSLPRFKFLLVCSVERDYCSLIRTVLDMMVERNLGKDGLLNKESLDMLADIQLLNRAIKRRNTKMAETLIHYSVNPSTRNFIFLPSIAGPGDITPLHLAASTSSSDDMIDALTNDPQEIGLSCWNTLVDATGQTPFSYAAMRDNHSYNTLVARKLADKRNGQISLNIENGIDQIGLSKRLSSELKRSCNTCASVALKYQRKVSGSRRLFPTPIIHSMLAVATVCVCVCVFMHAFPMVRQGSHFSWGGLDYGSI

>OsSPL1

MSSGLKKKGLEWDLNDWRWDSNLFLATPSNASPSKCSRRELGRAEGEIDFGVVDKRRRVSPEDDDGEECINAATTNGDDGQISGQRGRSSEDEMPRQGTCSSSGPCCQVDGCTVNLSSARDYNKRHKVCEVHTKSGVVRIKNVEHRFCQQCSRFHFLQEFDEGKKSCRSRLAQHNRRRRKVQVQAGVDVNSLHENHSLSNTLLLLLKQLSGLDSSGPSEQINGPNYLTNLVKNLAALAGTQRNQDMLKNANSAAIASHTGNYVAKGNSLHDSRPHIPVGTESTAEEPTVERRVQNFDLNDAYVEGDENRTDKIVFKLFGKEPNDFPSDLRAQILSWLSNCPSDIESYIRPGCIILTIYMRLPNWMWDKLAADPAHWIQKLISLSTDTLWRTGWMYARVQDYLTLSCNGNLMLASPWQPAIGNKHQILFITPIAVACSSTANFSVKGLNIAQPTTKLLCIFGGKYLIQEATEKLLDDTKMQRGPQCLTFSCSFPSTSGRGFIEVEDLDQSSLSFPFVVAEEDVCSEIRTLEHLLNLVSFDDTLVEKNDLLASRDRALNFLHEFGWFLQRSHIRATSETPKDCTEGFPAARFRWLLSFAVDREFCAVIKKLLDTLFQGGVDLDVQSTVEFVLKQDLVFVAVNKRSKPLIDFLLTYTTSSAPMDGTESAAPAQFLFTPDIAGPSDITPLHIAATYSDTAGVLDALTDDPQQLGIKAWKNARDATGLTPEDYARKRGHESYIEMVQNKIDSRLPKAHVSVTISSTTSTTDFTEKHASQSKTTDQTAFDVEKGQQISTKPPLSCRQCLPELAYRHHLNRFLSTRPAVLSLVAIAAVCVCVGLIMQGPPHIGGMRGPFRWNSLRSGPK*

>OsSPL2

MDWDAKMPSWDLGTVVGPSGGGGGGGGGGGALDLKLGAPTSWKTTTTVSAASAAPAAVAPPPPPPASSSSSAAAAGKRARAGQGQQAAVPACSVEGCAADLSKCVRDYHRRHKVCEAHSKTAVVTVAGQQQRFCQQCSRFHLLGEFDEEKRSCRKRLDGHNKRRRKPQPDPLNPGNLFANHHGAARFTSYPQIFSTAASMSPQETKWPANVVKTEAADVFQEPYYHALHLNGAGAAAAASIFHHGGNKARKHHFPFLTADHGGGAAAASPLFGCQPFTITPSSESRSSSSSRHSNGKMFAHDGGLDNCALSLLSDNPTPTAQITIPQPLFAGGGQYGGGGGGDVSLTGLSYVRMAGKDTSILAKSATTTATTATTPTTTSAQLQYHGYYHHHVSADQGSSDAAIQALPFSSW*

>OsSPL3

MGSFGMDWNQKSSVLWDWENMPPIGNSANENPKNVMLAESKLAGVGVDIGHESGHSSGGTFSSSSEIGYGSSKSSISASIDSPSKVGNTIELNFASAEEHDKNMDKGKSKVDDTGTSRSPVVAANRVEPLIGLKLGKRTYFEDVCGGQNVKSSPSGVSVATPSPGLAKKVKVAQQNTQNPHCQVEGCNVDLSSAKPYHRKHRVCEPHSKTLKVIVAGLERRFCQQCSRFHGLAEFDQKKRSCRRRLHDHNARRRKPQPEAISLSSSRLSTLLYGDARQQASFLFGQAPYGQMGSCASSWDNPVPGGFKFTATKAPWSRPTIAAGVDGTHVSNQQASGNVLPHGAHHSFDGLMAFKETNAKVLNQGMEASAVASGSARGPDFEHALSLLSIDSVGAANLQPGSQIHPGVTAIAGTSNPVMMPSPAIWQGGLSLDQQAQFQAFDRLGNDDDEDHLQLPKPSYDNSHYDQMN*

>OsSPL4

MDWMPPPKPTSPRSPPLLWDWADAAVPGSSSGEVSAAAAAAAAHPGRRRKEKRGRAEEGGGGGGEVRCQVEGCGVELVGVKDYHRKHRVCEAHSKFPRVVVAGQERRFCQQCSRFHALSEFDQKKRSCRRRLYDHNARRRKPQTDVFSYASARPPSSLLFDDNRQISFVWNKAPLSHVRPFAISPWESSSEVGTTDGHIYLDKSHISKSLPAFNTDIDELLPMKDFSAATIWMFFGVLFIRTAQESCMSFH*

>OsSPL5

MAVPAAAVAADVVDFGYAAPMPPPYVGFDPAGMGGERQLFQHGGACHGLYDGGLDFSAAAAFQEAATLGVGLPGGNLLQSLAPPAAAAATPSSLQMPMMMSLPGLPATAADVYPFGGGGFVKREDGPVLDVVGGGGGGRIGLNLGRRTYFSPADVLAVDRLLLRSRLGGMGMEMGMGMGVLGLGLAAAAHHHQPPRCQAEGCKADLSAAKHYHRRHKVCDFHAKAAAVLAAGKQQRFCQQCSRFHVLAEFDEAKRSCRKRLTEHNRRRRKPTAGGQSSKDSPPPPPSKKGTDASIASSYTSCDHHKAAASTTTASGVSCLQELADHHDVGGGHQAAMAAAPPPTLSLAALPPQEEDDEDEDGGLGNVLMMQQHHQRRRLQHDGDGDDDVAAAAAHHHLMRSLARQQQQHRHSSGCSNNNDGDDDDHNNNNNILSCSSASDQQNSSNNNNMHFFEVDFI*

>OsSPL6

MEAARVGAQSRHLYGGGLGEPDMDRRDKRLFGWDLNDWRWDSDRFVATPVPAAEASGLALNSSPSSSEEAGAASVRNVNARGDSDKRKRVVVIDDDDVEDDELVENGGGSLSLRIGGDAVAHGAGVGGGADEEDRNGKKIRVQGGSPSGPACQVEGCTADLTGVRDYHRRHKVCEMHAKATTAVVGNTVQRFCQQCSRFHPLQEFDEGKRSCRRRLAGHNRRRRKTRPEVAVGGSAFTEDKISSYLLLGLLGVCANLNADNAEHLRGQELISGLLRNLGAVAKSLDPKELCKLLEACQSMQDGSNAGTSETANALVNTAVAEAAGPSNSKMPFVNGDQCGLASSSVVPVQSKSPTVATPDPPACKFKDFDLNDTYGGMEGFEDGYEGSPTPAFKTTDSPNCPSWMHQDSTQSPPQTSGNSDSTSAQSLSSSNGDAQCRTDKIVFKLFEKVPSDLPPVLRSQILGWLSSSPTDIESYIRPGCIILTVYLRLVESAWKELSDNMSSYLDKLLNSSTGNFWASGLVFVMVRHQIAFMHNGQLMLDRPLANSAHHYCKILCVRPIAAPFSTKVNFRVEGLNLVSDSSRLICSFEGSCIFQEDTDNIVDDVEHDDIEYLNFCCPLPSSRGRGFVEVEDGGFSNGFFPFIIAEQDICSEVCELESIFESSSHEQADDDNARNQALEFLNELGWLLHRANIISKQDKVPLASFNIWRFRNLGIFAMEREWCAVTKLLLDFLFTGLVDIGSQSPEEVVLSENLLHAAVRMKSAQMVRFLLGYKPNESLKRTAETFLFRPDAQGPSKFTPLHIAAATDDAEDVLDALTNDPGLVGINTWRNARDGAGFTPEDYARQRGNDAYLNMVEKKINKHLGKGHVVLGVPSSIHPVITDGVKPGEVSLEIGMTVPPPAPSCNACSRQALMYPNSTARTFLYRPAMLTVMGIAVICVCVGLLLHTCPKVYAAPTFRWELLERGPM*

>OsSPL7

MEGNGCGGSGATPRGVVGMHWAPVVTSPPSPQPPFLPPAPCRPDVQMQQQGGLTCLKLGKRPCFWGGDGAGQVAQGSGGGGGGGGGGSADQGKRKEKAATAVPVVPRCQVEGCDITLQGVKEYHRRHKVCEVHAKAPRVVVHGTEQRFCQQCSRFHVLAEFDDAKKSCRRRLAGHNERRRRSNASEAMARGSAHPHGMPVLGHGFPPYGLPTSSAGALSLLSSARATGPWLMPTPDISARSSAALDELIAENRAALLSWQFFSDRQPPPAGRPTGRSPGSETAGGWHAHLQARPPPPGAGGQHENQSGHVTLDLMQATTAAGGSGAPFRPVPARPPKEGGDAGCTSDAWTPSPMEGARVV*

>OsSPL8

MMNVPSAAAASSCDDFGYNATPPPPPSLLPIMDQDGGGGSIQRDHHQHHNHQQLGYNLEPSSLALLPPSNAAAAAAHHATIAHASPHDLLQFYPTSHYLAAAGGAGGGGNPYSHFTAAAAAGSTFQSYYQQPPQDAPEYYFPTLVSSAEENMASFAATQLGLNLGYRTYFPPRGGYTYGHHPPRCQAEGCKADLSSAKRYHRRHKVCEHHSKAPVVVTAGGLHQRFCQQCSRFHLLDEFDDAKKSCRKRLADHNRRRRKSKPSDGEHSGEKRRAQANKSAATKDKAGSSSKNAGIGDGFETQLLGGAHMSKDQDQAMDLGEVVKEAVDPKGKASMQQQQQQAHHGIHQQSHQQHGFPFPSSSGSCLFPQSQGAVSSTDTSNIAQVQEPSLAFHQQHHQHSNILQLGQAMFDLDFDH*

>OsSPL9

MDAPGGGGGGGGGGGGVDAGEPVWDWGNLLDFAVHDDDSLVLPWGDDSIGIEADPAEAALLPPAPSPQPAEAEAEAAGPASLPSSMQAEGSKRRVRKRDPRLVCPNYLAGRVPCACPEIDEMAAALEVEDVATELLAGARKKPKGAGRGSGAAVGGSGGGASRGTPAEMKCQVPGCEADIRELKGYHRRHRVCLRCAHAAAVMLDGVQKRYCQQCGKFHILLDFDEDKRSCRRKLERHNKRRRRKPDSKGILEKDIDDQLDFSADGSGDGELREENIDVTTSETLETVLSNKVLDRETPVGSDDVLSSPTCAQPSLQIDQSKSLVTFAASVEACLGTKQENTKLTNSPVHDTKSTYSSSCPTGRVSFKLYDWNPAEFPRRLRHQIFEWLSSMPVELEGYIRPGCTILTVFVAMPQHMWDKLSEDTGNLVKSLVNAPNSLLLGKGAFFIHVNNMIFQVLKDGATLTSTRLEVQSPRIHYVHPSWFEAGKPIDLILCGSSLDQPKFRSLVSFDGLYLKHDCRRILSHETFDCIGSGEHILDSQHEIFRINITTSKLDTHGPAFVEVENMFGLSNFVPILVGSKHLCSELEQIHDALCGSSDISSDPCELRGLRQTAMLGFLIDIGWLIRKPSIDEFQNLLSLANIQRWICMMKFLIQNDFINVLEIIVNSLDNIIGSELLSNLEKGRLENHVTEFLGYVSEARNIVDNRPKYDKQRQVDTRWAGDYAPNQPKLGISVPLAESTGTSGEHDLHSTNAASGEEENMPLVTKALPHRQCCHPETSARWLNAASIGAFPGGAMRMRLATTVVIGAVVCFAACVVLFHPHRVGVLAAPVKRYLSRNYSS*

>OsSPL10

MMSGRMNAAGDESPFPFGAMQAPGPGAYVGFDHGAAAVAAAAAAAQRAGMLQHHHHHMYDGLDFAAAMQFGGGQDAPPHPQLLALPPSMAAPPPPPMPMPLQMPMTMPMPGDVYPALGIVKREGGGGGQDAAAGRIGLNLGRRTYFSPGDMLAVDRLLMRSRLGGVFGLGFGGAHHQPPRCQAEGCKADLSGAKHYHRRHKVCEYHAKASVVAASGKQQRFCQQCSRFHVLTEFDEAKRSCRKRLAEHNRRRRKPAAAATTAVAAAKDAAAAPVAAGKKPSGGAATSYTGDNKNVVSMSAAKSPISSNTSVISCLPEQGKHAAAAARPTALTLGGAPPHESSAPQIGAMLHHHHHHQQDHMQVSSLVHINGGGGGGSNNILSCSSVCSSALPSTATNGEVSDQNNDNSHNNGGNNNNMHLFEVDFM*

>OsSPL11

MECNPVSSTTSSSLLWDWDATASAEPPPPPGKRGGRDSSSASASAKRGRSAAAAGDAAAVAAEAPRCQVEGCGLELGGYKEYYRKHRVCEPHTKCLRVVVAGQDRRFCQQCSRFHAPSEFDQEKRSCRRRLSDHNARRRKPQTDVFAFGSGTLPRSLFDDRQQISFAWDNNAPLNHANTTSSSSWTSDLQLSQVMDISKRSRKAGADSANIRLSNALPTLCHDTNELLPIKGADASETASKLDGALDVQRALSLLSASSRGLTDPGHQTSSIIQFTNSNQNSTLPSVPSEGNSNVPFWVDGQHQAVEPQVFQFTMDTGNTVFPDLERIKPSYESSMFGLNQIH*

>OsSPL12

MASFGMNWNQKSPVFWDWENPAPFGPNTMENPKSIPHPEPRGVVVAAANHGSTNSSGGTFTSSSELANGSSKSSLSASFDSSSKLGNSLEFRFASVKGHGKNMCKDGEAGRVEDSGTSPAVAVSHGEPVIGLKLGKRTYFENVCGGQNVKSSSAASGVTCPSTVVKKMKVSQQSTQSSYCQVEGCKVDLSSAREYHRKHKVCEAHSKAPKVIVSGLERRFCQQCSRFHGLAEFDQKKKSCRRRLSDHNARRRKPQQEAISFGSSRLATMFYDARQQTDIYFGQSPFGQVRSNAISSCDNLGGFKFTEAKLPWMKPMKTIGLEDLNFSTLQMPGNVVSHTVHHHDFDGLIPFKGNTPKVLNQGVDPACAVVSSNSNGAPDLRRALSLLSSDSWGPADVQAGSQVHPGGVMPPLAVAAATVTAPTNPVSVMHALHPSTGGGGFWQDGDDPPPLDHASQAQAFMHPGNGSSSGYGHLH*

>OsSPL13

MDRKDKARKNFSSSSSSSAASMAALAAAAAAGDGGAALPSPMEEDKKPRLVASSLAPVAGGGGGGSSSSAAVAAGASSSSSSSSVAAAARRGAGRAGGGAPSGGGGGPRCQVERCGVDLSEAGRYNRRHKVCQTHSKEPVVLVAGLRQRFCQQCSRFHELTEFDDAKRSCRRRLAGHNERRRKSAADTAHGENCRHADQDAGRSHQGTGNPPFQIR*

>OsSPL14

MEMASGGGAAAAAGGGVGGSGGGGGGGDEHRQLHGLKFGKKIYFEDAAAAAGGGGTGSGSGSASAAPPSSSSKAAGGGRGGGGKNKGKGVAAAAPPPPPPPPRCQVEGCGADLSGIKNYYCRHKVCFMHSKAPRVVVAGLEQRFCQQCSRFHLLPEFDQGKRSCRRRLAGHNERRRRPQTPLASRYGRLAASVGEHRRFRSFTLDFSYPRVPSSVRNAWPAIQPGDRISGGIQWHRNVAPHGHSSAVAGYGANTYSGQGSSSSGPPVFAGPNLPPGGCLAGVGAATDSSCALSLLSTQPWDTTTHSAAASHNQAAAMSTTTSFDGNPVAPSAMAGSYMAPSPWTGSRGHEGGGRSVAHQLPHEVSLDEVHPGPSHHAHFSGELELALQGNGPAPAPRIDPGSGSTFDQTSNTMDWSL*

>OsSPL15

MQREVGPQVAPPMFLHQIQPLPPHATAAKKRGNPWPAAAVAAAEAKGGGNWNPRMWDWDSRALTAKPSSDALRVNAGLSHHQQQQQQSPPAAAKAAEALRQGGGGSGGLNLQLGLREDAATPMDVSPAATTVSSSPSPPASSAPAQEPVVRPSKRVRSGSPGSASGGGGGGGGGGNSGGGGGSYPMCQVDDCRADLTNAKDYHRRHKVCEIHGKTTKALVGNQMQRFCQQCSRFHPLSEFDEGKRSCRRRLAGHNRRRRKTQPTDVASQLLLPGNQENAANRTQDIVNLITVIARLQGSNVGKLPSIPPIPDKDNLVQIISKINSINNGNSASKSPPSEAVDLNASHSQQQDSVQRTTNGFEKQTNGLDKQTNGFDKQADGFDKQAVPSTMDLLAVLSTALATSNPDSNTSQSQGSSDSSGNNKSKSQSTEPANVVNSHEKSIRVFSATRKNDALERSPEMYKQPDQETPPYLSLRLFGSTEEDVPCKMDTANKYLSSESSNPLDERSPSSSPPVTHKFFPIRSVDEDARIADYGEDIATVEVSTSRAWRAPPLELFKDSERPIENGSPPNPAYQSCYTSTSCSDHSPSTSNSDGQDRTGRIIFKLFGKEPSTIPGNLRGEIVNWLKHSPNEMEGYIRPGCLVLSMYLSMPAIAWDELEENLLQRVNTLVQGSDLDFWRKGRFLVRTDAQLVSYKDGATRLSKSWRTWNTPELTFVSPIAVVGGRKTSLILKGRNLTIPGTQIHCTSTGKYISKEVLCSAYPGTIYDDSGVETFDLPGEPHLILGRYFIEVENRFRGNSFPVIIANSSVCQELRSLEAELEGSQFVDGSSDDQAHDARRLKPKDEVLHFLNELGWLFQKAAASTSAEKSDSSGLDLMYFSTARFRYLLLFSSERDWCSLTKTLLEILAKRSLASDELSQETLEMLSEIHLLNRAVKRKSSHMARLLVQFVVVCPDDSKLYPFLPNVAGPGGLTPLHLAASIEDAVDIVDALTDDPQQIGLSCWHSALDDDGQSPETYAKLRNNNAYNELVAQKLVDRKNNQVTIMVGKEEIHMDQSGNVGEKNKSAIQALQIRSCNQCAILDAGLLRRPMHSRGLLARPYIHSMLAIAAVCVCVCVFMRALLRFNSGRSFKWERLDFGTI*

>OsSPL16

MEWDLKMPPAASWELADELENSGGGGVPAAVSSSSAAVGGGVNAGGGGRQECSVDLKLGGLGEFGGGGAQPRVAVAGEPAKGKGPAAAATGAAAAASSAPAKRPRGAAAAGQQQCPSCAVDGCKEDLSKCRDYHRRHKVCEAHSKTPLVVVSGREMRFCQQCSRFHLLQEFDEAKRSCRKRLDGHNRRRRKPQPDPMNSASYLASQQGARFSPFATPRPEASWTGMIKTEESPYYTHHQIPLGISSRQQHFVGSTSDGGRRFPFLQEGEISFGTGAGAGGVPMDQAAAAAAASVCQPLLKTVAPPPPPHGGGGSGGGKMFSDGGLTQVLDSDCALSLLSAPANSTAIDVGGGRVVVQPTEHIPMAQPLISGLQFGGGGGSSAWFAARPHHQAATGAAATAVVVSTAGFSCPVVESEQLNTVLSSNDNEMNYNGMFHVGGEGSSDGTSSSLPFSWQ*

>OsSPL17

MATGGSGGGGGGGGGGDDVHGLKFGKKIYFEQDAAASASAAAVESSSTSSGGGGKKGKGVAAAAAPPPPLPPRCQVEGCGVDLSGVKPYYCRHKVCYMHAKEPIVVVAGLEQRFCQQCSRCSVHMVRFHQLPEFDQEKKSCRRRLAGHNERRRKPTPGPLSSRYGRLAASFHEEPGRSRSFVVDFSYPRVPSSVRDAWPAIQPSDRMSGSIQWQGGHELHPHRSAVAGYSDHHAFSSHGGSAAGAPMLHHPAFELTSGGCLAGVATDSSCALSLLSTQPWDTTQSTSSHNRSPPMSSTASAFGGGNNPVSPSVMASNYMAASPGWNSSSRGHDGARNVHLPPPHGVVLNEVPPGSVHHGHFSGELELALQGGAPSNRPEAEHGSGSGAFSHSTNAMNWSL*

>OsSPL18

MDWDLKMPVSWDLAELEHNAVPNMAAAASAAEPGIAAVAASRGAPGRPECSVDLKLGGLGEFGAADALKEPAAAAKAPVSSAAAAASVAKVPPSTSTLKRPRGGGGGGGGQCPSCAVDGCKADLSKHRDYHRRHKVCEPHSKTPVVVVSGREMRFCQQCSRFHLLGEFDEAKRSCRKRLDGHNRRRRKPQADSMSSGSFMTSQQGTRFASFTPPRPEPSWPGIIKSEETPYYSHHHHPHPVMTSRQPHFVGSPSSATTAAFSPKEGRRFPFLHEGDQISFGGGGGAAAAATLEISVCQTTVVAPPPPESSSSNKMFSSDGLTTATTTTTTAHHHHHHHQVLDSDCALSLLSSPANSSSVDVSRMVQPSPAAAAGAEHHHHHQIPMAQPLVPNLQQQFGGSSPWFASSPAAAAVAGGGFACPSMDSEQQQQQQLNAVLVPGSNENEMNYHGMFHVGGEGSSDGTSPSLPFSWQ*

>OsSPL19

MEWAAAATKAASWGMAVAAAAAADDAGPTMLSFAGPSSSSSSPDAAAAAAAAAAAALHDFSVRARPAAAAPATRRARGGSGGGGGGGGGAEACSVDGCRSDLSRCRDYHRRHKVCEAHAKTPVVVVAGQEQRFCQQCSRFHNLAEFDDGKKSCRKRLDGHNRRRRKPQHDALNPRSFLPYHQANQFSVYPQTFPIADQNADALMRPLDRHPPFSISFSGTFREPKQFPFMQDGGSGLGAARHDLLRPFSSPEDGANITTTRSACNGVPHGLDPECALSLLSSSLHPSPAAGISSATAPPQFAPSSFSRIAASSQAVTTAFASDGGSVAGDHVLVPAVTYEDPSQAMPFSWQV*

>MtSPL1

MGERLGAENYHFYGVGGSSDLSGMGKRSREWNLNDWRWDGDLFIASRVNQVQAESLRVGQQFFPLGSGIPVVGGSSNTSSSCSEEGDLEKGNKEGEKKRRVIVLEDDGLNDKAGALSLNLAGHVSPVVERDGKKSRGAGGTSNRAVCQVEDCGADLSRGKDYHRRHKVCEMHSKASRALVGNAMQRFCQQCSRFHILEEFDEGKRSCRRRLAGHNKRRRKTNQEAVPNGSPTNDDQTSSYLLISLLKILSNMHSDRSDQPTDQDLLTHLLRSLASQNDEQGSKNLSNLLREQENLLREGGSSRNSGMVSALFSNGSQGSPTVITQHQPVSMNQMQQEMVHTHDVRTSDHQLISSIKPSISNSPPAYSETRDSSGQTKMNNFDLNDIYVDSDDGTEDLERLPVSTNLATSSVDYPWTQQDSHQSSPAQTSGNSDSASAQSPSSSSGEAQSRTDRIVFKLFGKEPNEFPLVLRAQILDWLSQSPTDIESYIRPGCIVLTIYLRQAEAVWEELCCDLTSSLIKLLDVSDDTFWKTGWVHIRVQHQMAFIFNGQVVIDTSLPFRSNNYSKIWTVSPIAVPASKRAQFSVKGVNLMRPATRLMCALEGKYLVCEDAHESTDQYSEELDELQCIQFSCSVPVSNGRGFIEIEDQGLSSSFFPFIVAEEDVCTEIRVLEPLLESSETDPDIEGTGKIKAKSQAMDFIHEMGWLLHRSQLKYRMVNLNSGVDLFPLQRFTWLMEFSMDHDWCAVVKKLLNLLLDETVNKGDHPTLYQALSEMGLLHRAVRRNSKQLVELLLRYVPDNTSDELGPEDKALVGGKNHSYLFRPDAVGPAGLTPLHIAAGKDGSEDVLDALTNDPCMVGIEAWKNARDSTGSTPEDYARLRGHYTYIHLVQKKINKTQGAAHVVVEIPSNMTESNKNPKQNESFTSLEIGKAEVRRSQGNCKLCDTKISCRTAVGRSMVYRPAMLSMVAIAAVCVCVALLFKSSPEVLYMFRPFRWESLDFGTS*

>MtSPL2

MDFGGNMFCLNNRDQSNTNNNGNSNGFTWCSSGSTTTGTAWNMNTFNVNEVNNSVSAANRTEAGLANALMYLPQNEGGGRQYQSAYGGGVSRGHMMPDPHLTCLKLGKRHYFEDVSGGGGVMGEKKGKGGYCGGGKTAAVGGYTAVTRCQVDGCNVALMNAKEYHRRHKVCEMHSKAPKVVVLGLEQRFCQQCSRFHVVSEFDDSKRSCRRRLAGHNERRRKSSHDSVGRNSSQGGCALSLLSSRTDSWLSPADLSVRCSAALSELIAENRASIMARQYVSDRDWHLQHHAVEDYKEIQSESNYFPQHMFPQTH*

>MtSPL3A

MDGSWGEGKRIYEYREENEYEVEVEIEEEEDVSYGDDEKRKRVVTDHLYNKKGSKAGGSVTPSCQVDNCNADLSAAKQYHKRHKVCENHSKAHSVLISELQQRFCQQCSRFHEVSEFDDLKRSCRRRLAGHNERRRKSASEYH*

>MtSPL3B

METRRSEGKRSLKYKEDHEEEEEEEEDTDFEEEEDGRRKRVVTDLYSKRSSKKAGSSNIPPCCQVENCDADLSEAKQYHRRHKVCEYHAKAPAVHIAGLQQRFCQQCSRFHGLSEFDDAKRSCRRRLAGHNERRRKSAIDYQGE*

>MtSPL4

MEGKQTSVEKVWKDIIFEEIEVEEEEEGGVEDDEKKKGVCVSGRKEPSRGGRFSSPICQVESCGADLTFSKRYHRRHKVCEVHSKASVVVVAGMRQRFCQQCSRFHELAEFDESKRSCRRRLARHNERRRKSTAGTCTEGSSTGQKNNDGDWRQIHMNITSSSGHDFLNFR*

>MtSPL5

MEARSIERKRTLMMEKMRRNNMVEEEMENEVEEEEGDVSLTEEEKKKGVGGGRRGGGGGVSPPCCQAERCGADLTDAKRYHRRHKVCEFHSKAPVVVVAGMRQRFCQQCSRFHDLVEFDESKRSCRRRLAGHNERRRKTNPETANEGSSHSKGQHQPKETQCRIQMNLPGSSGYKSFNIR*

>MtSPL6A

MESWSFDSRDKGYVSNDTLGSRTKGSILGWDLKTPSSFLPQQNIENDNNNHGFEELGFHGMLGKQLSNVVDDDDDVVVGSKIVTSNSFVMATPNAFSEREQQHFNSKHSNSIGDTNGSNSLIDLKLGRFGDHRDGIGTPFSKGTTILSSSGSSTPSKRVRSSAIHSQIAYCQVYGCNKDLSSCKDYHKRHKVCEVHSKTSKVIVNGIEQRFCQQCSRFHLLAEFDDGKRSCRKRLAGHNERRRKPQVGIHSGRAGRLLQSYGDSRFQGTMLTSASFICQDILPGEVFSSEKCGNSNWWRPIKAEGGTGFRPLSSIPITNAHPQSRTLFPSYNNKQFPFLQENGATSATGSIFCENNSQYPPIQGGSNSGSQSLFQDTSLGSEDFNVFETESAVQGLSGVSDGGCALSLLSSQSQNSSSQSTGIPMPGHLVIPSSHSHYSMSHVSESGVSDRFPSELNHTDGSHLSPILISNNNEIAHFELVDGVFQGSDFVNVKDRLSCEDGTTIDLLQLSSQLQRVEHQRRALQVKRENDSSCSLQIT*

>MtSPL6B

MAALQQVKRKVEKGKVYRGGRSSGVVRTLGDAAAPADQYVAPSQGTRFLHYFNISVTSIQFVVMESWSYIPEERSYLFSDEMDFSLDAFMRSRKPLVEWENKSSCNFERDGFNSDREVVKSMEFVDLGFPMESSRTVRGSVQTSSCELDSNNSSKRENSSVHVIAFDSSFAEEDSEAKHLSSRVESKNHDSSLIDLKLGRLVDCRGGGSDRDGKTFTSESMPIHQNVLAKRSRTSSLPALAPVCQVYGCNMDLSSSKDYHKRHKVCDVHSKTAKVIVNGVEQRFCQQCSRFHLVAEFDDGKRSCRRRLAGHNERRRKPQFDYMTSKQHKILQSYQGTNNFSFQDIFQSGILFPEKHDQISQSGHIKLEEDPICSPQLEAPATHGHELSSHALSLLSAQSQNPLHQAAGNPSATSSTFFRDIRTQDRGDQVSETPLMTSSMNKHERNESFPCGINSKENIKSEHGGTVDLFQLSSNLQRVEKQRNSVLVKWENEDCCFSNV*

>MtSPL6C

MESWSYISEEKGSEELGFCEMFGKQYSDDFNFIGNVPSKKLDGKFDDPNEFSVKGDTNFKLSNSVVESNGWDSLIDLKLGRFDDHGNASIDVAFSKGAAPILSSCESSTPPKRVRVHSMTAYCQVYGCNKDLSSCKDYHKRHKVCEVHSKTPIVIVNGIEQRFCQQCSRFHLLSEFDDGKRSCRKRLAGHNERRRKPQGIHSANSGRLFQPFGDIGFQGTKPPAASFLCPEVFQSGFWRPMKAEHEAGFRHLSSVPVTNGHLQSRSPFPSYNEKQFPFLHENVATSTTGSKFSENNSHYAHAIGYASLGNEDFNVFHTAPSIQGLSGISDSCALSLLSSQSNNTSSQSSEIPFDNPLVIPSSHSHVYSIRSQATSSSRVSDRFLSKLNPADGSHLSPILVSDNNDIVNFEMEDGIFHDSDFVNARDCLSCEDDATIDLLQLSSQLQRVEHHRQSLQVKKEFDSSCTLRIT*

>MtSPL7

MESKSQPILPPMDPPEDLSSVWDLSYLLDFDDDIPQLPPLPNPNPTPEENERIRKRDPRLTCSNFLAGQVPCACPELDALLEDNGLPGKKRARTARLIFSELKGYHRRHRVCLRCANAATVVLDGDVKRYCQQCGKFHVLSDFDEGKRSCRRKLERHNTRRRRKAVDSAVGVDNEVQTVTQNDDSNCDGELGIDYSNLSRENIEKRALQDHEEEPVVNGSSTPETQNINGDSVVSFVASAETQANIGKDVSDPSKSPSYCDNKSDYSSMCQTGRVSFKLYDWNPAEFPRRLRLQIFQWLASMPVELEGYIRPGCTILTIFIAMPNIMWINLLKDPMYYVRDLAAPRNMLSGRGTALIHLNDMIFRVMKDGISVTKVEVNMQAPRLHYIHPTCFEAGKPMEFFACGSNLLQPKFRLLVSFYGKYLKCEYCAPSPHNSAEDNISCAFDNQLYKICVPHIEENLLGPAFIEVENESGLSNFIPVLIGDKEICTELKILQQKLDASLLSKQFRSASGSSICSSCEAFVHIHTSSSDLLVDIAWLLKDPTSENFDRMVSASQIQRYCYLLDFLICNDSTIILGKILPNLISITKSMKSNISDVDMDQLLKGMCNARDAICRKGGGIVLNSKMEGFKPAQCSSQNAKLSVVEVNSQGIQFRADAELGVLSSLASDEKNQKIPLLKRDIIMNMEELPKRCDHQNLTRGFLRSRPTTFVLVSLVVCLAVCVSVFHHGRVNELAVSIRRCLFNH*

>MtSPL8

MLDYEWGNPSNIMLTGNEDNSGAAATDQAHRQIFDHYASQAMLSDNYLNGPGGGPTIDINSGDFTHHHNQFSPHNQQHHNIHQSFFDPRAFHGGSSTASSYPPPQPQPPPSMLSLDPLPGHGHSPGFLLVPKSEDVNRPIDFVGSRLGLNLGGRTYFSSEDDFVTRLYRRSRPPEPGSTGSSNSPRCQAEGCNADLSQAKHYHRRHKVCEFHSKAATVVAAGLTQRFCQQCSRFHLLSEFDNGKRSCRKRLADHNRRRRKTQHPNTQDIHKSHNTLDSSATRSPPESGTQSTSSVTVAVSPPDYFRQRSYQTPSPSTTSSSMFFSTG*

>MtSPL9

MSSGFVPSPSSPPNSSTEFLDGLKFGQKIYFEDATSTVVVPASQTKPNAIGVGGSSSSSSSSGMKRGNNHPPRCQVEGCKVDLSGAKAYYSRHKVCCMHSKSPSVVVSGLEQRFCQQCSRFHQLPEFDQGKRSCRRRLAGHNERRRKPQPSSFLTSRSPKLSSPTFGNNGKGNNDFLMECASYQKISMGNALSTPRSAKPVPGNQATQFTWQNNSSTTSDFFLQGTNFHAPPTRHLPIMDNYNEVTDSSCALSLLSNQTWCSRNTTPPSVEMNNLLNFNGSLTTQSSQVVPDYQLQTNASSWFLKGVDSRNCLSPEAVPDLGLGQNSQAIHSDLHGELDVSQGKRHYWPL*

>MtSPL10A

MDLNAISPSSQWDWEHQSFFNAKATEYPKLQPPSWSIEQDREINVGLFDTPGGSGIFGSASRSSKSASNSSSLNRNNSKTCIFACGNSHTPELSSVSGEPLLTLLGKRVYIEDVCPKSDSKNLSFSRDLASSLSIGKKCKFNSQNLQFPRCQVEGCGLDLSSAKDYHRKRRVCESHSKSPMVVIDGMVRRFCQQCGRFHNLAEFDEKKRSCREQLSRHNARRRKHQPEALQSSQSVLSSRCDGKQQMSPFANSKTATNLAWQNMQNSKLPQAKDFLLKPAKDNANTQGTVTMFSDDSIVHFTSKVLATKSIDRGVEDFITVSDTNDEQDFICALSLLSTNPWDSYATKSMSLEHSNRPTSAVQAVTHPMSQRMPLA*

>MtSPL10B

MEWNVKSPGQWDWENLFFLNSKAAETHRLQSTDWSMEEDREINVGMLIPSGGSGYSVSKLMHASSSRSSKSASNNSSSNEDSKTSMLTQEGSPDNSTGKKESSKGDPIETSPAAEPLLTLKLGKRLYFEDVSTGSHSKKASSSAVPLLCGKKGKSSSQNMLNPSCQVEGCGLDLSFAKDYHRKHRICDSHSKSPVVVVAGLERRFCQQCSRFHDLSEFDDKKRSCRRRLSDHNARRRKPQPEAVKLNPSALSSSPYDGRQAMGPFAFPKNTSNLAWPDMPNSKLPQTKDFMLKTPKNFNKIVTMLSDDSSGHFISKGKGTNIAVPGLEDPNTLSDPSATQDVNRALSLLSTNSWGAYDTKPPSFVHSNRTTGTPQYATAQRSPFSSPEYWHTDQHQASSSACISFSGYDNSNRYQDFQLFSEPYESSFPCNQLD*

>MtSPL11A

MDLNAKSPSSQWEWDHLPLPDTKATENKKLQPPNWSIKLDRENNVWLFDTPTGSGYSGSELIPGSTSGSSKSDSISSSPTRDISKTCKFAFESSHDDSNGKIELPKEGPIEASNAPMLSCVSSEPLLNLKLGKRMKFEDVSSLVFGRKCKSNDQNLQCPPLCQVEGCGLDLSSAKHYYRKRRVCVDHSKSPMVVIDGLERRFCQQCSRFHDLFEFDGKKKSCRRRLSQHNARRRNHSRKTAQSSQSALSSSPCDGKQQMNPFAYSKTAINLALQNIQNSEFPRIKDFPLKYAKVNIGTPSFVTMLSDDSDVHFTSKVLETKSINAGVEDFISFSDTDATEDFTCASSLLSTSPWDSYATKSVSLVHSDRTTSTAQAITHTTSQRIPLTSSEYWHGDLDQHVDSSMWISNSRL*

>MtSPL11B

MDLNAISPSSQWEWDHLPLLDTKATENQKLQPPNWTMELDPEINFGLYDTPVGSGSSGSDLIHGSAFNAPKFSCVSGEPLLNPRLGKRMYFEEVCPESDSKNLSFSRDLMSSLILEKKCKSNGQNLQCPPHCQVEGCGLNLSSAKDYHCKRRVCESHAKSPMVVIDGLERRFCQQCSRFHDLFEFDGKKKSCRRQLSNHNARRRKYHRQAVQSSQSALSYSRSDGKQQMSPFTNSKTATNLAWQNMHNSKLPQAKDFLLKPSKDNIDTPSTVTMLSHDSNFHFTSKILATKSVNPGVEDFMNFSDTNATQDFTCALSLLSTNQWDSYATKSISQEHSNRPTSPFQATTHAMSQCIPLASSEPCCGHHDQYVNSNIWISNSADSNHFQKFQLFREPYEL*

>MtSPL12

MDADFEGKNQHLYEAVVPEMKGAVGRGSKDWDLNDWRWDGDLFTAKQLNSVPTDCRNRQFLPEIRENVDVSNNLISGEGSRELEKRRRGFGGEGLEMNDDFGSLNLNLGGQVYPIMDGEEKSGKKTKIAPVPTSNRAVCQVEDCRADLSNAKDYHRRHKVCDVHSKASKALVGSVMQRFCQQCSRFHVLQEFDEGKRSCRRRLAGHNKRRRKTHPDTAVVNGGSPNEERGSSYLLMSLLRILSNMHSNGPDHTRNLDGLSHLIGNLTSLAGTFNGRNIASLLEGPQELVKAGTSGAAQNVPNSNPNGAEPSRPDSSIEMTNGLIHQDPPESRLQCATVPANHLTQKCIPSSSVGVGCLKPPLIPQSSNLVPSRGSLPPRPVATETTVGRNRLCNIDLNNVYDDGQDYVENPENSNPPLALGVESRDHSSFVQYESLKSSPPQTSRNSDSTSTQSPSSSSGEGQSRTDRIVFKLFGKDPNDIPHVLRSQVLSWLSNSPTEIESYIRPGCIILTICLRLENSAWDELCYNLGPSLRKLLAASNDSLWRTGWIYTRVQHSVAILYNGQLVLDVPSRLGSPQNCQILCIKPLAVSANEDVKFTVKGLSLFLSSARLLCALEGKYLVEDMCYDLIDGADAAIGHHELQTLSFSCHIPNMTGRGFIEVEDNSLSSCSFPFIVAEQEICSEICSLETIIEAAETADDIQIKAKLMEEKTRAMNFVQEMGWLLHRIRIKFRLGPMTPVQDRFHLNRYTWLVGFSMDHDWCAVMKKLLDTIFEGEVDTGEHISAELALLNMGLLHKAVKRNCRPMVELLLNFVPVKASDGGDSKEMQVNKVPDGFLFRPDTVGPAGLTPLHVAASMNGYETVLDALTDDPGMVGIEAWKSAKDNTGLTPNDYASLRGHYSYIQLVQRKTSKSSQTQHVLDIPGTLVDGNTMKQSDGHKSSKVLSLHTEKIATTAIPNHCGICQQKLAYGSVGGMRRALVYRPAMLSMVAIAAVCVCVALLFKSSPRVSYVFQPFSWESLDYGSI*

>MtSPL13A

MEWSNLKAPSWDLTEMDQGTLNNIETMDGSIRRFGDYRTKGDFSVDLKLGHVGNSSTESGLTKSKDVGGFTKMTSSSSSTSGSSKRARAINNGTQIVSCLVDGCQADLSNCRDYHRRHKVCELHSKTAQVTIGGHKQRFCQQCSRFHALEEFDEGKRSCRKRLDGHNRRRRKPQPEPITRSSSFLSNYQGTQLLPFSSSHVYPSTAMMNPTWVGGDVRLHSHNQHQQMHHLVDKQDLFLGTTSSTSSYKEGKQVSFIQNNQPTAAATMNNQPLPASSMSRTFSRTNPFSERYKMFCDNNTSCALSLLSSPVPQTHDHPENGLNQMVNTHSSFMQPLGLSLHDHSLGSVDSVLGSNDQSGHCSSMYNMGSSESHSQGNNNEASPLFPFQWD*

>MtSPL13B

MEWNLKAPSWDLGGIEEATLPNIETMEESNRFGVYKMKGEFSVDLKLGQVGNSATDQSPLPLSNDAVVVSKIATPTSSSGSSKRARAMNNNTTLTVSCLVDGCNSDLSNCRDYHRRHKVCELHSKTPEVTICGLKQRFCQQCSRFHSLEQFDERKRSCRKRLDGHNRRRRKPQPEPITRPAGSFLSNYQGTQLLPFSSSTTMVNSTWSSGLISSCESGRLHINNQHQQVHVVDKQDHFLGSTATTYEGKQLQFLHNDNNNPSLHNETAPLLRTSSKMFCDSLATSVHESPCALSLLSSSQTRIPDNGLNQMVQQPHSMSHMQPLGLSLHGNNSFESMEGVLVPNGSESDHCSSLYNMGSDGSQGNDAPQLFPYQWE*

>MtSPL13C

MDWDWKEFAWDPSGFDEELKNEGDSMDLRLGDQASDSLEKSVHDSGKEDSKAVSSSLSPSGSLKRSRLQNGSQSMICSVDGCNSDLSDCREYHKRHRVCEKHSKTPVVLVGGKQQRFCQQCSRFHSLAEFDDVKRSCRKRLDGHNKRRRKPQPPSLFMAAEKFMYNYKGPRILHFGSPQTYVNPIMRNIWPATSITEAESGYDHQRLLYRIDKHKQDKGHPHWQEILPKSGDVNKAAPGTPISHPIRGAVGSSAGEKGGRKLSSDGKPGSFDSGCALYLLSTLQTQSSELSLMQSSINSPTQSSSGTLHFDATKEYSYSGKVKDKPNGPVFVLDGNTTNLHCNGMLQMGPNGLIENDNSMTLPFFWE*

>MtSPL14

MEKVAPPLLPLHPPMLSSHQFYDSSNTKKRDLLSSYDVVHIPNDNWNPKEWNWDSIRFMTAKSTTVEPQQVEESLNLNLGSTGLVRPNKRIRSGSPTSASYPMCQVDNCKEDLSKAKDYHRRHKVCEAHSKASKALLGNQMQRFCQQCSRFHPLVEFDEGKRSCRRRLAGHNRRRRKTQPDEVAVGGSPPLNQVAANLEIFNLLTAIADGSQGKFEERRSQVPDKEQLVQILNRIPLPADLTAKLLDVGNNLNAKNDNVQMETSPSYHHRDDQLNNAPPAPLTKDFLAVLSTTPSTPARNGGNGSTSSADHMRERSSGSSQSPNDDSDCQEDVRVKLPLQLFGSSPENDSPSKLPSSRKYFSSESSNPVDERTPSSSPPVVEMNFGLQGGIRGFNSNCISTGFGGNANKETSQSHSCTTIPLDLFKGSKSNNMIQQSSSVQSVPFKAGYASSGSDYSPPSLNSDTQDRTGRIMFKLFDKHPSHFPGTLRTQIYNWLSTRPSDLESYIRPGCVVLSIYASMSSAAWVQLEENFLQRVDSLIHNSDSDFWRNGRFLVYSGSQLASHKDGRIRMCKPWGTWRSPELISVSPLAIVGGQETSISLKGRNLSAPGTKIHCTGADCYTSSEVIGSGDPGMVYDEIKLSGFEVQNTSPSVLGRCFIEVENGFKGNSFPVIIANASICKELRPLESEFDEEEKMCDAISEEHEHHFGRPKSRDEALHFLNELGWLFQRERFSNVHEVPDYSLDRFKFVLTFSVERNCCMLVKTLLDMLVDKHFEGEGLSTGSVEMLKAIQLLNRAVKRKCTSMVDLLINYSITSKNDTSKKYVFPPNLEGPGGITPLHLAASTTDSEGVIDSLTNDPQEIGLKCWETLADENGQTPHAYAMMRNNHSYNMLVARKCSDRQRSEVSVRIDNEIEHPSLGIELMQKRINQVKRVGDSCSKCAIAEVRAKRRFSGSRSWLHGPFIHSMLAVAAVCVCVCVLFRGTPYVGSVSPFRWENLNYGTM*

>MtSPL15

MDSGGNSSSEESSLNGLKFGQRIYFEDTALTAASAAAASTTIAAGSPSSSGSKKGRGGSVQHSQPPRCQVEGCKLDLTDAKAYYSRHKVCSMHSKSPTVTVSGLQQRFCQQCSRFHQLAEFDQGKRSCRRRLAGHNERRRKPPPSSLLTSRFARLSSSVFGNSDRGGSFLMEFASNPKHSLRNSPGNQTTAIGWPWPGNTESPSSNLFLQGSVGGTSFPGARHPPEETYTGVTDSNCALSLLSNQTWGSQNTEPSPGLNNMLNFNGTPMTQLGTSSHGVAMHQIPNNYEVVPDLGRGHILHPLGSQHSGELDLLQQGRRHYMDVEHSRAYESSQWSL*

>MtSPL16

MEAFQLYGNGGGSSDLRAMGKNSKEWDLNNWKWDSHLFIATSKLTPVPEHRQFLPIPVGGGGGGGGSNSNSSSSCSEQLDLGICQVKEGERKRRVIVVEDELGLGLNKEGGNLSLNLGGGVATWEGNNGKKSRVAGGGSSSRAFCQVEDCRADLNNAKDYHRRHKVCEIHSKASKALVGNAMQRFCQQCSRFHLLQEFDEGKRSCRRRLAGHNKRRRKTNQDAVPNGSSPNDDQTSSYLLISLLKILSNMQPDRSNQTADQDLLTHLLRSLANQNGEQGGRNLSNLLREPENLLKEGSLSGKSEMVSTLVTNGSQGSPTVTVQNQTVSISEIQHQVMHSHDARVADQQTTFSAKPGVSNSPPAYSEARDSTAGQTKMNDFDLNDIYIDSDDGIEDIERLPVTTNLGASSLDYPWMQQDSHQSSPPQTSGNSDSASAQSPSSSTGETQNRTDRIVFKLFGKGPGDFPLVLKAQILDWLSHSPTDIEGYIRPGCVVLTIYLRQAEVVWEELCFDLTSSLNRLLGVSDDDFWRTGWVHIRVQHQMAFIFNGQIVIDTPLPFRSNNYGKILSVSPIAIPSSKTAQFSVKGINLTRPATRLLCALEGNYLDCEDTDEPMDQCSKDLDELQCIQFSCSVPAMNGRGFIEIEDQGLSSSFFPFIVVEEDVCSEICVLEPLLESSDTYPDNEGAGKIQAKNQAMDFIHEMGWLLHRRQIKSSVRLNSSMDLFPLDRFKWLMEFSVDHDWCAVVKKLLNLMLDGTVSTGDHTSLYLALSELGLLHRAVRRNSRQLVELLLRFVPQNISDKLGPEDKALVNGENQNFLFRPDAVGPAGLTPLHIAAGKDGSEDVLDALTNDPCMVGIEAWNSARDSTGSTPEDYARLRGHYTYIHLVQKKINKSQGGAHVVVDIPSIPTKFDTSQKKDESCTTFQIGNAEVKKVRKDCKLCDHKLSCRTAVRKSFVYRPAMLSMVAIAAVCVCVALLFKSSPEVLYIFRPFRWESLDYGTS*

>MtmiR156B

CTCCTCAGACAACAACAAGTTTCTCATTTGTTCCATCGAAAAGGAACATTTAGCTTCTTTGTTGTAGAATTTACAGAGATATGGTTTGTTTATTATTTTCTTTTCCTCCTAACCCTCCATCCCCTATTTCAGAAATAATTTATAAGGGTTAGCATAGTTTGATGCAATACTTCCAAGCTTAAGGAAGGGTGATCGACGCATATCAGATTCAATCGAGGGTAAGGGAGGTGACAGAAGAGAGTGAGCACACATGGTACTTTCGTGTATGATGTTTCATTCTCGAAGCTATGTGTGCTCACTCTCTATCTGTCACCCCATCACCATCTTTTTCTTTAATTCAATTCAATCCCTTTAATTATATTGTTTTCTAATACTATGCATGTCTGTTTATGCATCATATCTCATATTATGCATCTCAGCTACATTTTCTTTTCATATCATATATCATTCTTTATATATACGAGGTGCTTTTTAGGCTGATCTGATCAGTTTCATGAAAATCATTTCTGATTTAGGTCTGCCAAGTTTTGTAAAAATGGTATGTGAACGCCAGCGCCGCTGTGGCCTTAACATCCATGTTTTTTTATGTCTTTGTGAGCAAATTAATGCAGGCATTAGTTGTATTTGACTGCGATTTTCCATAATATGATGCAATTATAGCGTAATTGCGACCACAATTTAAAATTAAGATTAATTTATGTATGAAAGACATGTGTGTGTCTAGATGCAGAAGCTATTTTCACAATTATGAATATTGACTCTAATAATTAATTAATGAATTTGGATCTTATACTCTCAGTTTAAAGCTTTGACAAAAATCCTACCACCAG
