# Supplementary figures and images for: Genome-wide characterization of SPL family in Medicago truncatula reveals the novel roles of miR156/SPL module in spiky pod development
Source: BMC Genomics. 2019 Jul 5;20:552. doi: 10.1186/s12864-019-5937-1 (PMC6612136; doi:10.1186/s12864-019-5937-1)

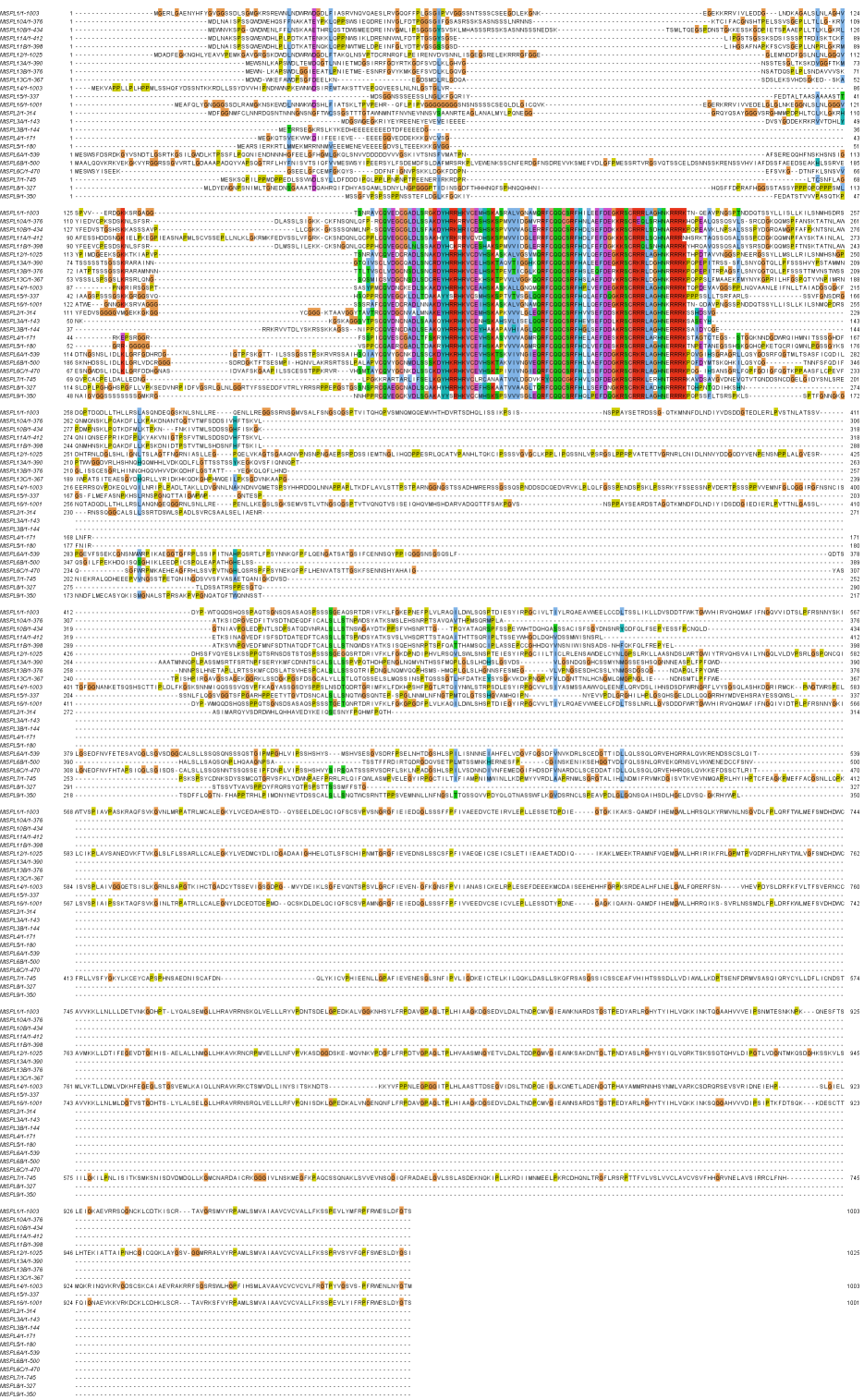

Supplement: Supplementary file 2 — Multiple amino acid sequences alignment of MtSPL proteins using full-length amino acid sequences. Sequences were aligned using Jalview software. (DOCX 575 kb) [file 12864_2019_5937_MOESM2_ESM.docx]

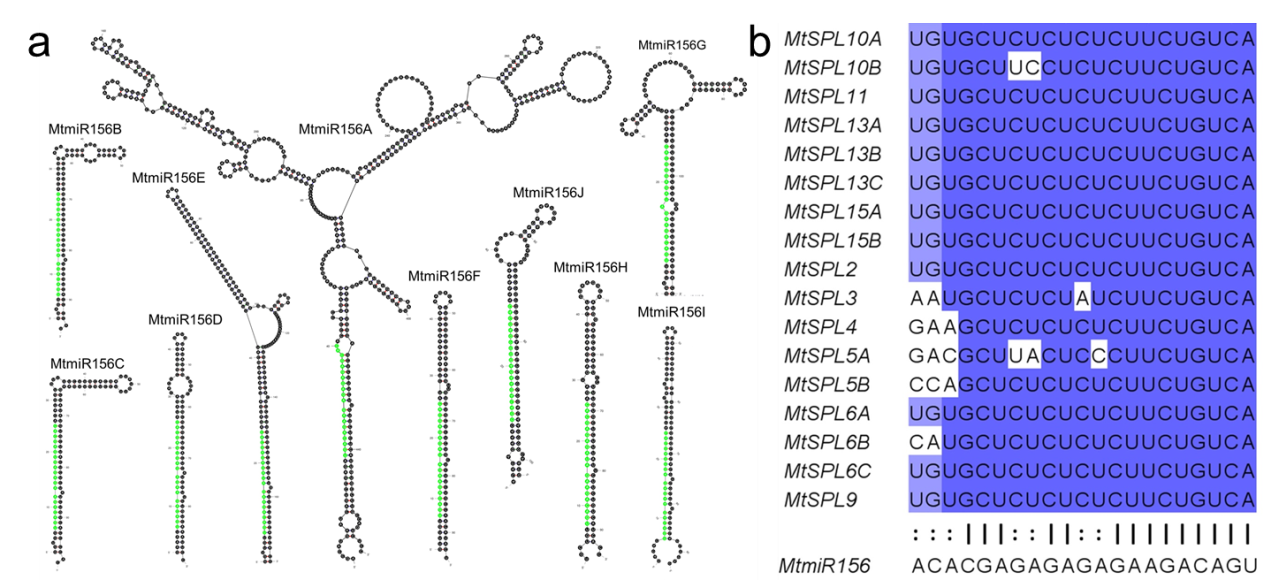

Supplement: Supplementary file 3 — Secondary structure of MtmiR156 and regulation of MtSPLs by MtmiR156. a RNA secondary structures of the MtmiR156A-MtmiR156A J were predicted by the online mfold Web Server. The nucleotides with light green color in stem-loop structures indicate the mature MtmiR156 sequences. b Multiple MtSPL genes were regulated by MtmiR156. The deoxyribonucleotide with shaded color indicates the conserved sequences targeted by MtmiR156. (DOCX 548 kb) [file 12864_2019_5937_MOESM3_ESM.docx]

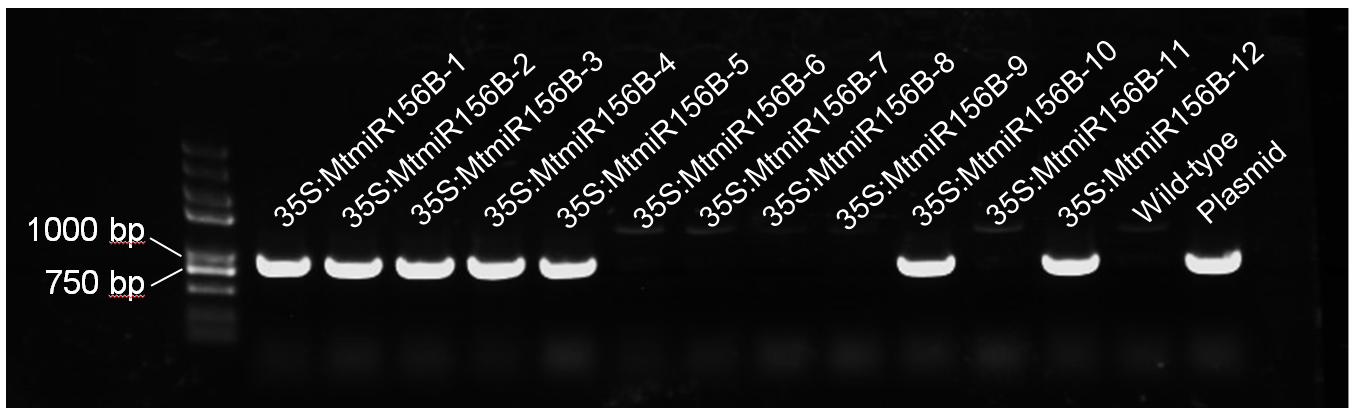

Supplement: Supplementary file 4 — Molecular characterization of MtmiR156B overexpression lines. PCR analysis was performed using primer pair 35S-F/MtmiR156B-R for regenerated transgenic plants together with the positive control (35S:MtmiR156B), and negative control (Wild-type). (DOCX 168 kb) [file 12864_2019_5937_MOESM4_ESM.docx]
