# Supplementary material for: Genome-wide characterization of SPL family in Medicago truncatula reveals the novel roles of miR156/SPL module in spiky pod development
Source: BMC Genomics. 2019 Jul 5;20:552. doi: 10.1186/s12864-019-5937-1 (PMC6612136; doi:10.1186/s12864-019-5937-1)
Supplement: Supplementary file 5 — Primers used in this study. (DOCX 23 kb) [file 12864_2019_5937_MOESM5_ESM.docx]

| MtSPL1-qRT-F | TGCTCTGACTAATGATCCTTGCA | For qRT-PCR analysis of *MtSPL1* |
| --- | --- | --- |
| MtSPL1-qRT-R | CCTGTACTGTCACGGGCATTC |  |
| MtSPL2-qRT-F | TCAGCGCTCACCTTTTTCG | For qRT-PCR analysis of *MtSPL2* |
| MtSPL2-qRT-R | GGAGCTGGCCTGATGTTGAT |  |
| MtSPL3-qRT-F | CAAAGCACCTGCCGTACACA | For qRT-PCR analysis of *MtSPL3* |
| MtSPL3-qRT-R | TCTGCTACATTGCTGACAAAACC |  |
| MtSPL4-qRT-F | TTCTAAGGCTCATTCCGTACTCATT | For qRT-PCR analysis of *MtSPL4* |
| MtSPL4-qRT-R | CCTGCTACATTGCTGGCAAA |  |
| MtSPL5A-qRT-F | CAAAGCATCTGTTGTGGTGGTT | For qRT-PCR analysis of *MtSPL5A* |
| MtSPL5A-qRT-R | CCTGCTACATTGTTGGCAAAAC |  |
| MtSPL5B-qRT-F | CATCGCCGCCATAAAGTGT | For qRT-PCR analysis of *MtSPL5B* |
| MtSPL5B-qRT-R | CCCTGCAACCACCACAACA |  |
| MtSPL6A-qRT-F | GCTGAGTTCGACGATGGTAAGC | For qRT-PCR analysis of *MtSPL6A* |
| MtSPL6A-qRT-R | CCTTCGGCGCTCATTGTG |  |
| MtSPL6B-qRT-F | CGTCACAACTTCAGCGTGTT | For qRT-PCR analysis of *MtSPL6B* |
| MtSPL6B-qRT-R | TTTTCTCGCTTCACCTGCAA |  |
| MtSPL6C-qRT-F | CAAGCCACCAGCAGCATCT | For qRT-PCR analysis of *MtSPL6C* |
| MtSPL6C-qRT-R | GGTCTCCAGAAACCACTTTGGA |  |
| MtSPL7-qRT-F | CCTCCGTTTGGGACCTCTCT | For qRT-PCR analysis of *MtSPL7* |
| MtSPL7-qRT-R | GCGGGAGTTGTGGAATATCG |  |
| MtSPL8-qRT-F | CAAGCCGAAGGTTGCAATG | For qRT-PCR analysis of *MtSPL8* |
| MtSPL8-qRT-R | GTGGCGGCGGTGGTAA |  |
| MtSPL9-qRT-F | TGGGTGTGCTCTCTCTCTTCTG | For qRT-PCR analysis of *MtSPL9* |
| MtSPL9-qRT-R | GAGGTCAGCCGGTGAAAGC |  |
| MtSPL10A-qRT-F | CGCCCGACCAGTCCTTT | For qRT-PCR analysis of *MtSPL10A* |
| MtSPL10A-qRT-R | GCAAGAGGAATGCACTGACTCA |  |
| MtSPL10B-qRT-F | ACGACGTCTTTCACAACACAATG | For qRT-PCR analysis of *MtSPL10B* |
| MtSPL10B-qRT-R | TGATTGGGCAGTTTTACGAGAA |  |
| MtSPL11-qRT-F | TGGTATGGTACGTCGATTTTGC | For qRT-PCR analysis of *MtSPL11* |
| MtSPL11-qRT-R | TTCATCGAACTCAGCCAGATTG |  |
| MtSPL12-qRT-F | ATACCATCATCAAAGACAGCTCAA | For qRT-PCR analysis of *MtSPL12* |
| MtSPL12-qRT-R | TGTGGCAGGGCGAGTCA |  |
| MtSPL13A-qRT-F | GCAGCAGCCACTATGAACAATC | For qRT-PCR analysis of *MtSPL13A* |
| MtSPL13A-qRT-R | CTGCTAAACGTGCGACTCATG |  |
| MtSPL13B-qRT-F | TTGGGAACTCTGCCACTGATC | For qRT-PCR analysis of *MtSPL13B* |
| MtSPL13B-qRT-R | TTTTGGAGACAACAACAGCATCAT |  |
| MtSPL13C-qRT-F | GGACATAATAAGCGCAGAAGGAA | For qRT-PCR analysis of *MtSPL13C* |
| MtSPL13C-qRT-R | GAACTTCTCAGCAGCCATGAAA |  |
| MtSPL14-qRT-F | GGATTTTCTTGCTGTTCTTTCCA | For qRT-PCR analysis of *MtSPL14* |
| MtSPL14-qRT-R | CGTTGCCGCCATTTCG |  |
| MtSPL15A-qRT-F | CAAAGCATCTGTTGTGGTGGTT | For qRT-PCR analysis of *MtSPL15A* |
| MtSPL15A-qRT-R | CCTGCTACATTGTTGGCAAAAC |  |
| MtSPL15B-qRT-F | GCCAACAGTGTAGCAGGTTTCAT | For qRT-PCR analysis of *MtSPL15B* |
| MtSPL15B-qRT-R | GCGGCAACTTCGTTTTCCT |  |
| MtSPL16-qRT-F | GCAGCAAGTATGAATGGCTATGA | For qRT-PCR analysis of *MtSPL16* |
| MtSPL16-qRT-R | CCAACCATTCCCGGATCA |  |
| MtmiR156B-qRT-F | GCTGTGGCCTTAACATCCATGT | For qRT-PCR analysis of *MtmiR156B* |
| MtmiR156B-qRT-R | AAATCGCAGTCAAATACAACTAATG |  |
| MtmiR156B-F | CACCCTCCTCAGACAACAACAAGTTT | For cloning of the *MtmiR156B* gene |
| MtmiR156B-R | CTGGTGGTAGGATTTTTGTCAA |  |
| MtUBI-qRT-F | CTGACAGCCCACTGAATTGTGA | For qRT-PCR analysis of *MtUBI* |
| MtUBI-qRT-R | TTTTGGCATTGCTGCAAGC |  |
